# Therapeutic Potential of a Water-Soluble Silver-Diclofenac Coordination Polymer on 3D Pancreatic Cancer Spheroids

**DOI:** 10.1021/acs.jmedchem.2c00535

**Published:** 2022-08-15

**Authors:** Sabina W. Jaros, Urszula K. Komarnicka, Agnieszka Kyzioł, Barbara Pucelik, Dmytro S. Nesterov, Alexander M. Kirillov, Piotr Smoleński

**Affiliations:** † Faculty of Chemistry, University of Wrocław, F. Joliot-Curie 14, 50-383 Wrocław, Poland; ‡ Faculty of Chemistry, Jagiellonian University, Gronostajowa 2, 30-387 Kraków, Poland; § Malopolska Centre of Biotechnology, Jagiellonian University, Gronostajowa 2, 30-387 Kraków, Poland; ∥ Centro de Química Estrutural, Institute of Molecular Sciences, Departamento de Engenharia Química, Instituto Superior Técnico, Universidade de Lisboa, Av. Rovisco Pais, 1049-001 Lisbon, Portugal

## Abstract

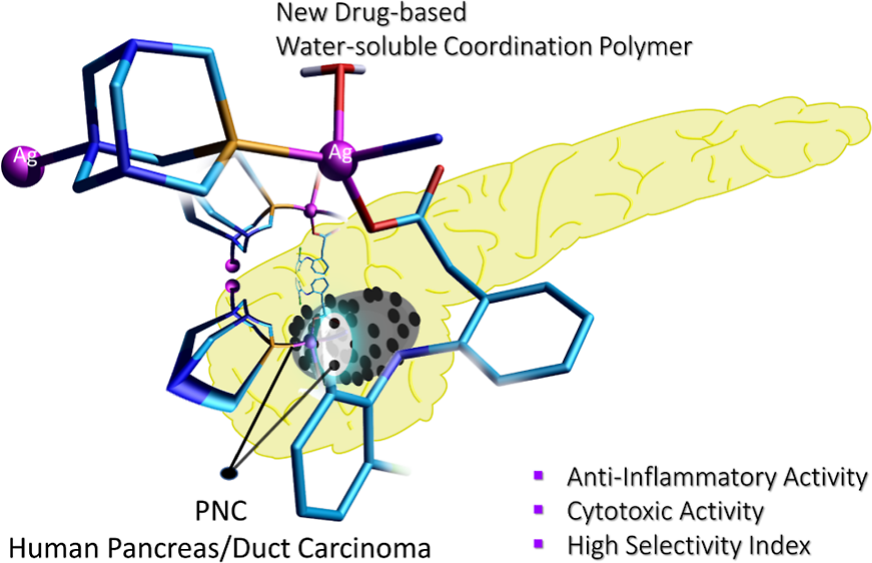

This work describes the traditional wet and green synthetic approaches, structural features, and extensive bioactivity study for a new coordination polymer [Ag(*μ*-PTA)(Df)(H_2_O)]_*n*_·3*n*H_2_O (**1**) that bears a silver(I) center, a 1,3,5-triaza-phosphaadamantane (PTA) linker, and a nonsteroidal anti-inflammatory drug, diclofenac (Df^–^). Compared to cisplatin, compound **1** exhibits both anti-inflammatory properties and very remarkable cytotoxicity toward various cancer cell lines with a high value of selectivity index. Additionally, the 3D model representing human pancreas/duct carcinoma (PANC-1) and human lung adenocarcinoma (A549) was designed and applied as a clear proof of the remarkable therapeutic potential of **1**. The obtained experimental data indicate that **1** induces an apoptotic pathway via reactive oxygen species generation, targeting mitochondria due to their membrane depolarization. This study broadens a group of bioactive metal–organic networks and highlights the significant potential of such compounds in developing advanced therapeutic solutions.

## 1 Introduction

Intensive research on the design of coordination polymers (CPs) with bioactivity has opened up a platform for advanced therapeutic materials.^1−4^ In particular, new drug carriers and delivery systems based on bioinspired CPs are a subject of high interest as potential anticancer agents.^5−8^ Immobilization of biorelevant metal ions and active pharmaceutical ingredients (APIs) into metal–organic networks paves the way toward more biocompatible systems with promising anticancer activity and possible synergic effect of different building blocks.

In particular, new therapeutic agents against pancreatic ductal adenocarcinoma (PDAC) are urgently needed.^9−12^ PDAC is one of the most insidious and resistant cell lines. A combination of factors such as delayed diagnosis at a metastatic stage, tumor location, and low effectiveness of available Pt-based chemotherapeutic solutions significantly limit treatment options proposed to pancreatic cancer patients. Statistically, over 97% of patients die within 6 months after diagnosis.^10−12^ Therefore, new chemotherapeutic agents and strategies based on metal–organic derivatives that address the demand for improved therapeutic efficacy and lower toxicity characteristics can be considered as promising alternatives to Pt-based anticancer agents.^1−3,6,13^

Among different bioactive metal ions and compounds, silver(I) derivatives are particularly interesting.^13−17^ Diverse coordination features, tunable bonding properties, and low toxicity make Ag(I) an attractive metal center for coordinating with endogenous APIs, mainly the derivatives of aminophenylacetic acid included in nonsteroidal anti-inflammatory drugs (NSAIDs).^18^ Diclofenac (HDf) and its sodium salt (NaDf) are widely used in clinical treatment as low solubility NSAIDs.^19^ Besides, diclofenac exhibits cytotoxic effects and induces apoptotic death of various cancer cells, including multidrug-resistant cells.^19^ The experimental results suggest that NSAIDs, especially those that act as very selective inhibitors of cyclooxygenase-2, exert promising properties in terms of their anticancer activity. Nonetheless, their use is associated with serious gastrointestinal toxicity.^20,21^

Thus, the combination of diclofenac with Ag(I) ions within a CP network driven by a water-soluble 1,3,5-triaza-7-phosphaadamantane linker (abbreviated as PTA) can lead to a plausible synergic bioactivity effect of these three different components on account of an appropriate silver coordination environment, lower toxicity, improved solubility, tunable degradability, and desired anticancer and anti-inflammatory properties associated with programmed release of active components.^22−25^ Besides, there are no reports on the delivery systems for APIs based on Ag-PTA coordination networks and their targeted anticancer therapy applications. Bearing all these points in mind, herein, we describe a new bioactive Ag(I) CP, [Ag(*μ*-PTA)(Df)(H_2_O)]_*n*_·3*n*H_2_O (**1**), which combines two types of organic building blocks, namely, a water-soluble PTA linker and a NSAID diclofenac.

## 2 Results and Discussion

### 2.1 Synthetic Methodology

**Scheme 1:**
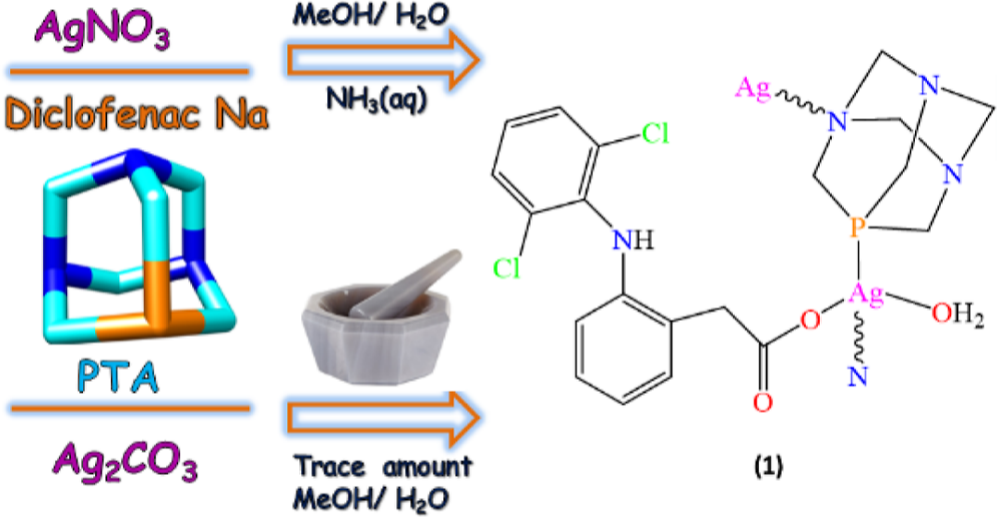
Simplified Synthetic Procedure for Compound **1**

[[Ag(*μ*-PTA)(Df)(H_2_O)]_*n*_·3*n*H_2_O] (**1**) was prepared (Scheme 1) using a conventional solution synthesis based on a self-assembly approach, as well as a mechanochemical liquid-assisted grinding (LAG)^26−29^ route. Both synthetic pathways for **1** are stoichiometric (AgNO_3_/PTA/NaDf: 1:1:1). The methanol/water medium self-assembly gives rise to monocrystals of **1** suitable for X-ray diffraction measurements. The LAG was applied as an alternative and cost-effective route for a “scale-up” synthesis of **1**. In this route, the reaction is initiated by mechanical action and a trace amount of solvent (water/methanol). A simple grinding of silver carbonate with PTA and NaDf produces a yellowish-white microcrystalline sample of **1**. The following methods were applied to characterize **1** comprehensively: X-ray crystallography, NMR, and Fourier-transform infrared spectroscopy (FTIR), elemental analysis, and ESI-MS(±). PXRD (powder X-ray diffraction) profiles and FTIR data for samples prepared by solution self-assembly and LAG procedures are in good agreement (see the Supporting Information). Compound **1** is >98% analytically pure on the basis of elemental analysis, as well as >95% crystalline pure phase as confirmed by PXRD analysis (Figure S1, Supporting Information).

### 2.2 Structural Features

**Figure 1:**
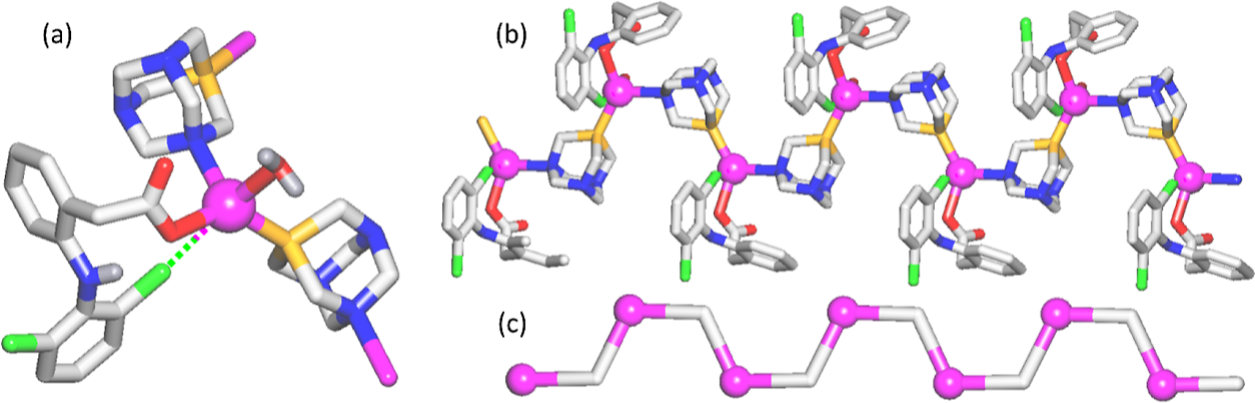
Crystal structure of **1**. (a) Connectivity of the Ag atom and ligands. (b) Tooth-like CP and (c) its simplified topological view (2C1 net). Additional details: (a) only NH and H_2_O hydrogen atoms are shown. The particular elements of the structural skeleton are color-coded as follows: silver (magenta), nitrogen (blue), phosphorus (orange), carbon (pale gray), oxygen (red), chlorine (green), and hydrogen (gray); (b,c) view down the *a* axis; (c) centroids of *μ*-PTA (gray) and silver (magenta).

The crystal structure of [Ag(*μ*-PTA)(Df)(H_2_O)]_*n*_·3*n*H_2_O (**1**) features the tooth-shaped 1D metal–organic chains assembled from Ag(I) centers, *μ*-PTA linkers, and terminal Df^–^ and H_2_O ligands (Figure 1). The coordination environment around the Ag atom is formed by P and N donors of two *μ*-PTA linkers [Ag–P 2.384(1), Ag–N 2.405(4) Å], O donors from Df^–^ and H_2_O ligands [Ag–O 2.303(1) and 2.543(3) Å, respectively], and a weakly bound Cl atom of the same Df^–^ moiety Ag–Cl 3.366(3) Å. The reported Ag–Cl distance is lower than the van der Waals radii sum for these two atoms, 3.47 Å. As a result, the coordination {AgPNO_2_Cl} geometry can be defined trigonal bipyramidal with distortions, wherein O (H_2_O) and Cl (Df^–^) atoms are in axial sites with a Cl–Ag–O angle of 170.6°. In **1**, the 1D chains feature a 2C1 topology and further interact with H_2_O molecules of crystallization to generate a complex 2D H-bonded network. Within this network, an unusual type of 2D H-bonded water layer with a **hcb** topology can be identified (Figure S2, Supporting Information).

An important feature of **1** concerns its stability and solubility (*S*_25 °C_ ∼ 0.5 mg mL^–1^) in water over a wide pH range with conservation of ligand environment around Ag(I) ions containing coordinated PTA and Df^–^ ligands (Figures S8–S21, S23, and S24; Table S1), as well as a negative lipophilicity parameter (log *P* = −0.61) in comparison to positive values for HDf (4.5) and NaDf (1.4). The solubility (*S*_25 °C_) equals ∼0.5 mg mL^–1^. There is a 400-fold decrease in water solubility at pH = 4 compared to the neutral conditions.^20^ The determination of log *P* is an important tool for studying the bioavailability of a potential drug. A slightly negative value for **1** shows its predominant hydrophilic properties (see the Supporting Information), which encouraged us to evaluate its bioactivity in an aqueous medium.^30,31^ CP **1** is also stable under pseudopharmacological conditions (50 or 5 mM sodium chloride in deuterated H_2_O/DMSO solutions, see the Supporting Information). Monitoring of the stability of **1** in the acid buffer (pH = 4.0 and 5.5) indicates that it can act under slightly acidic pH conditions common for cancer cells.^32^ ESI-MS data of the H_2_O/CH_3_OH solutions reveal the dominating [Ag_2_(PTA)_2_(Df)]^+^ (*m*/*z* 824, 100%) and [Ag(PTA)_2_]^+^ fragments (*m*/*z* 421) in MS(+) spectra and the [Ag(PTA)(Df)(H_2_O)_3_ – H]^−^ (*m*/*z* 613) fragment in the negative mode. A further fragmentation pattern of these ionic species is typical for Ag-PTA derivatives (see the Supporting Information, Figures S23 and S24).^23−25^ The species considered the most robust in solution according to ESI-MS/MS studies was observed at *m*/*z* 421 and is assigned to [Ag(PTA)_2_]^+^ ions. There is also a progressive cleavage of the PTA cage with preservation of silver–phosphorus bonding, with characteristic fragments at *m*/*z* 237, 223, 197, and 177. Fragmentation patterns of higher mass ions are also typical for Ag-PTA derivatives.^23−25^ However, in the case of cell medium, it is difficult to assess speciation as we are dealing with many additional factors that can cause other interaction products and pathways (e.g., under the influence of glucose, fructose, sulfide ions, etc.). Nevertheless, the performed tests indicate that there is no decomposition of **1** to give free silver ions under the conditions of the cell medium.

### 2.3 Cytotoxicity

The cytotoxicity of **1** along with the corresponding precursors (PTA, NaDf, and AgNO_3_) and cisplatin (reference drug) was evaluated on the following human cell lines: (1) cancer cell lines: MCF-7 (breast adenocarcinoma), A549 (lung adenocarcinoma), DU-145 (prostate carcinoma), and PANC-1 (pancreas/duct carcinoma) and (2) normal somatic cell lines: MRC5 (primary cells of pulmonary fibroblasts) and HaCat (keratinocytes). The cell cytotoxicity is concentration-dependent and increases on augmenting the concentration of **1**. Viability, evaluated by the 3-(4,5-dimethylthiazol-2-yl)-2,5-diphenyltetrazolium bromide (MTT) assay, permitted to determine the IC_50_ values after 72 h incubation with **1** versus untreated control cells (Table 1). Also, to exclude the effect of releasing free silver(I) ions, the toxicity of soluble Ag(I) salt was checked in vitro. These tests suggested that the silver(I) toxicity can be neglected in the concentration range that is predicted after the decomposition of the original tested compound (Figure S3, Supporting Information).

**Table 1:** IC_50_ Values (μM) for **1** and Various Control Compounds Determined for Selected Cell Lines after 72 h Incubation with **1** vs Untreated Control Cells

cell line	A549	MCF7	DU-145	PANC-1	MRC5	HaCat
**1**	62.3 ± 4.1	11.1 ± 2.1	41.6 ± 1.3	3.1 ± 0.2	87.1 ± 2.1	58.7 ± 3.01
PTA	1171.3 ± 14.7	1471.1 ± 14.4	1234.4 ± 21.9	1292.2 ± 52.6	867.2 ± 18.3	825.8 ± 8.76
NaDf	699.3 ± 7.1	978.2 ± 10.4	803.2 ± 14.7	801.3 ± 124.3	977.3 ± 5.9	1144.3 ± 18.3
cisplatin	>100	50.9 ± 7.6	>100	12.5 ± 1.3	31.5 ± 4.1	26.43 ± 2.7

Among all the investigated cancer cell lines, A549 cells are the most resistant against Ag(I) derivatives, while the most sensitive line is PANC-1. Notably, **1** exhibits significantly higher cytotoxicity than both ligand precursors (PTA, NaDf; Table 1). A cytotoxic effect of **1** toward all the tested cancer cell lines is also more prominent when compared to nonmalignant lines. CP **1** features a notably superior cytotoxic activity against PANC-1 cells (IC_50_ = 3.1 ± 0.2 μM) among all the tested cancer cell lines. The selectivity index (SI) is defined as a ratio between the IC_50_ values of nonmalignant and tumor cells. For example, the SI for PANC-1 attains 28.1 and 18.9 with regard to the MRC5 and HaCat normal cell lines, respectively.

**Figure 2:**
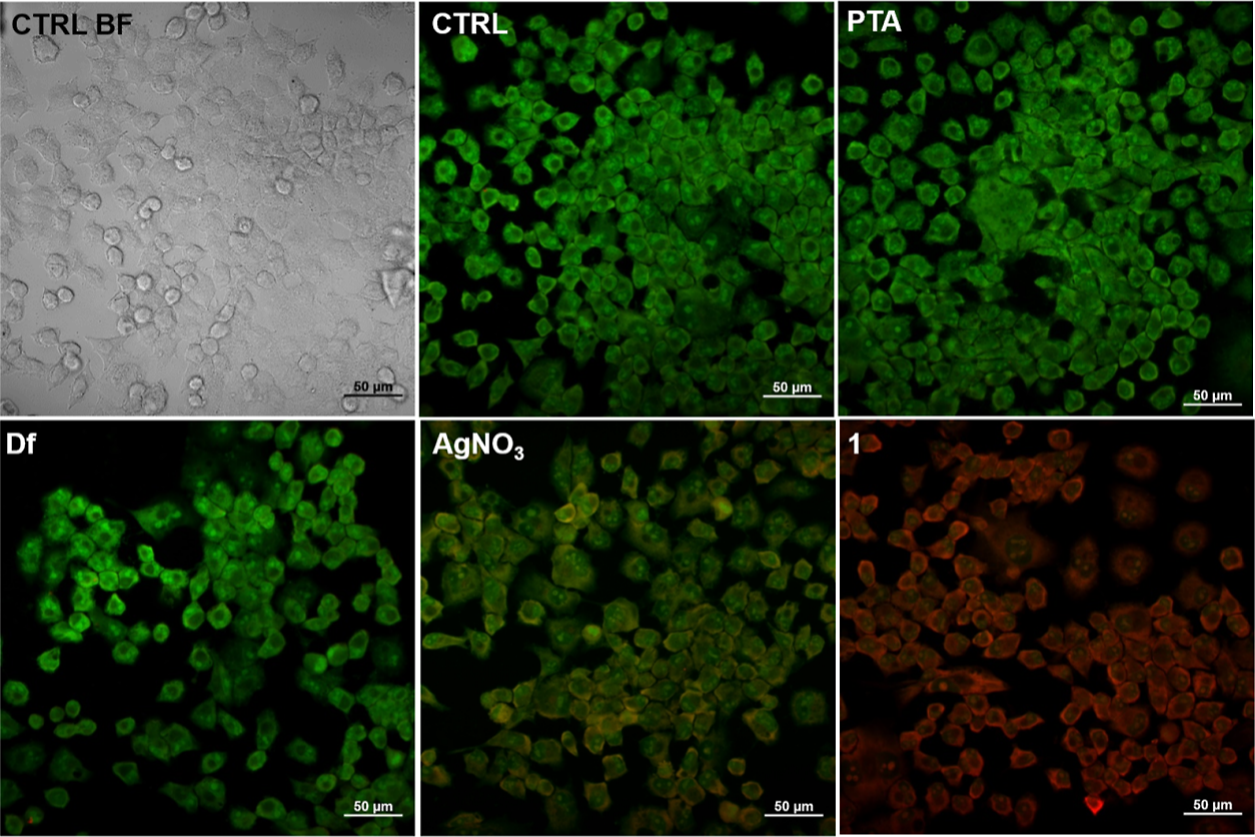
Bright-field images of PANC-1 cells: CTRL BF—untreated cells (bright field), CTRL—untreated cells stained with OA/PI, and images of AO/PI stained PANC-1 cells (72 h after treatment): **1** and PTA, NaDf (diclofenac), and AgNO_3_ at the respective concentrations based on the molar ratio of 1:1:1 (PTA/NaDf/AgNO_3_). Cells with normal morphology and intense green nuclei—viable, round red cells—dead. Scale bar: 50 μm.

**Figure 3:**
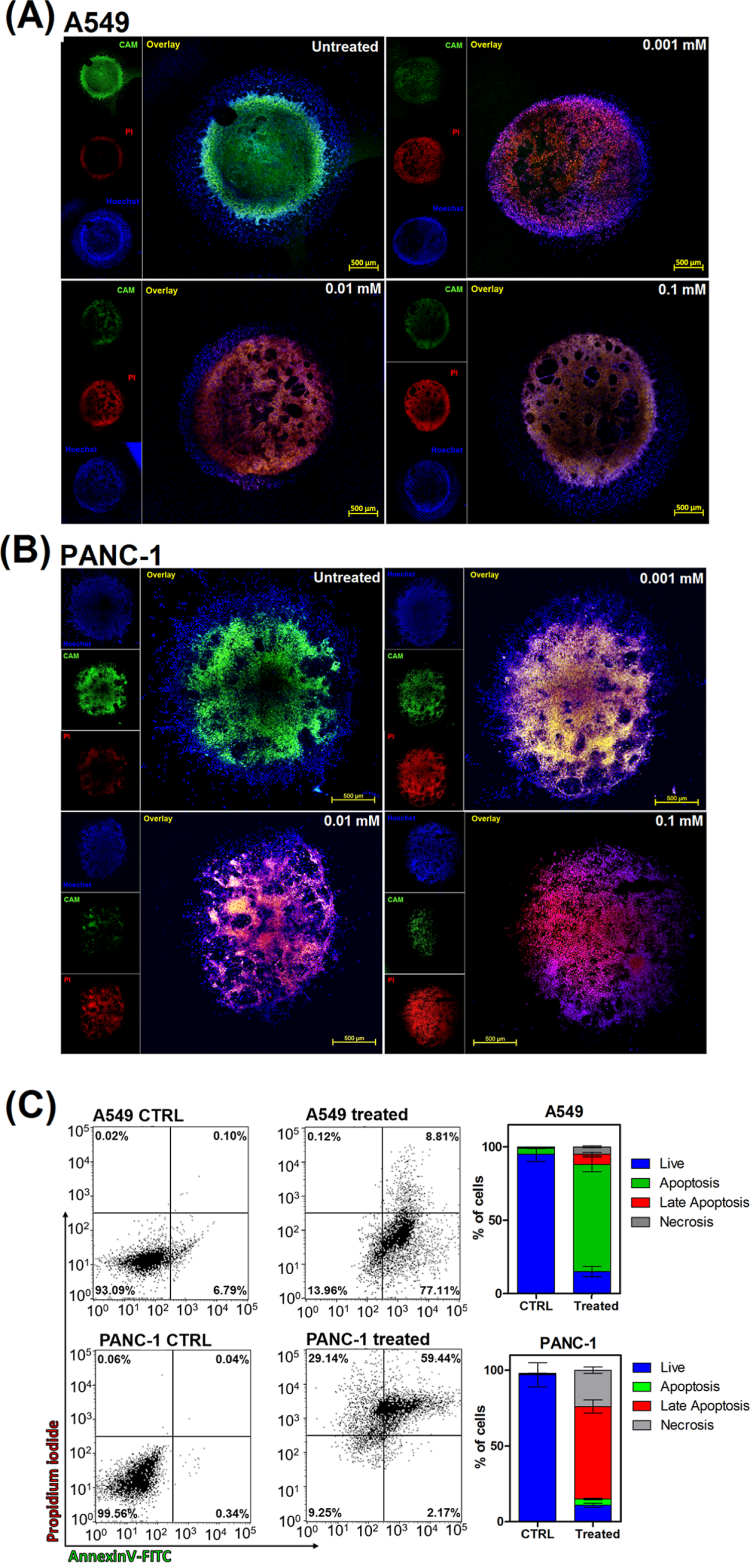
(A) A549 and (B) PANC-1 3D tumor spheroid model: untreated controls and assessment of the cytotoxicity of compound **1** (*C* = 0.1, 0.01, and 0.001 mM). PI: propidium iodide (red, death cells), CAM: calcein-AM (green, live cells), Hoechst (blue, nuclei); scale bar: 500 μm. (C) Cell death quantification in spheroids treated with **1** at 0.1 mM; these are presented as average values with standard deviation estimated from three independent tests.

Importantly, the activities of **1** toward all the tested malignant cell line types are significantly higher than those of cisplatin (in case of PANC-1 cells, the activity of **1** is 4-fold). In view of recent reports on tumor growth inhibition (caused by diclofenac for PANCO2—a murine type of pancreatic cancer cell line) by modulating the arginase activity and levels of VEGF,^33^ the effect observed for **1** may explain selectivity toward this type of tumor cells. The cytotoxicity of **1** determined via the MTT assay was further confirmed by acridine orange/propidium iodide (AO/PI) staining of PANC-1 cells after treating the cells at the IC_50_ concentration of **1** for 72 h (Figure 2). Although the MTT assay is a colorimetric method to count viable cells in the presence of cytotoxic agents, AO/PI staining also provides information on the possible cell death that can be distinguished between apoptotic and necrotic cells. AO/PI serve as fluorescent markers to visualize simultaneously both dead and live cell types. Acridine orange readily enters living cells and induces green fluorescence. PI has no permeability to living cells with intact cell membranes and thus is commonly used for the visualization of dying and dead cells with disrupted membrane integrity.^34^ In AO/PI staining, the untreated PANC-1 cells (Figure 3—CTRL) did not show any morphological changes, and only intact green-colored, viable cells were observed, indicating no signs of cell death. Similarly, the treatment with NaDf and PTA (Figure 3—PTA and Df) did not induce visible cytotoxicity. However, a moderate cytotoxic effect was observed upon treatment with AgNO_3_ (Figure 3—AgNO_3_), where double-positive AO+/PI+ cells (orange color) are visualized. Furthermore, after treatment with **1** (Figure 3—1), cell death was evident, and most of the PI-positive, necrotic cells were clearly seen with the red fluorescence.

The obtained experimental evidence is in good agreement with that of Altay et al.^35−38^ on significant cytotoxicity of silver coordination compounds with nonsteroidal drugs (e.g., NaDf) against the selected cancer cell lines in vitro with simultaneous low cytotoxicity of the ligands alone. Besides, Banti et al. established that diclofenac conjugated with biocides resulted in an enhanced synergistic antiproliferation effect, particularly on MCF-7 cells.^39^ All the above findings and our experimental data strongly indicate that, as a ligand, diclofenac significantly contributes to a potent anticancer activity of **1**.

### 2.4 Therapeutic Potential on 3D Spheroids

Three-dimensional spheroids represent recognized models in the development of novel antitumor agents, which feature a number of advantages if compared to the conventional 2D cell cultures that cannot always mimic the heterogeneity and complexity of clinically isolated tumor examples. Hence, there is a recent shift in studying 3D spheroid models with different tumor microenvironment characteristics.^40−42^ Herein, we designed and developed a 3D model (Figure 3) representing the tumor growth of human pancreas/duct carcinoma (PANC-1) and human lung adenocarcinoma (A549).

The activity of **1** against three-dimensional spheroids was simultaneously monitored by in situ dead/live fluorescence staining, resulting in the data on cytotoxicity and dead cell distribution. The A549 and PANC-1 spheroid cultures demonstrate a significant sensitivity to the chemotherapeutic agent **1** (Figure 3). Even at the lowest concentration of 1 μM, a significant reduction in cell viability was observed. After exposure to **1**, the integrity of spheroids appeared to be destroyed at the used concentration in the presence of dead cells at a 3D spheroid inner cores. Spheroid disruption clearly reflected a cell loss. Evident spheroid shedding and disruption, as well as dead cells (red fluorescence), were observed in both types of spheroids. Viable cells (green fluorescence) could still be detected after treatment (especially at the lowest concentration of **1**), but the signal from treated spheroids was significantly reduced compared to untreated control. Thus, these results showed that the efficacy of the drug tested in this study (compound **1**) was generally comparable in tumor spheroids, as indicated in monolayer culture. Moreover, for the understanding of the cell-death mechanism induced by **1** and the quantification of nonliving cells, the cell samples from the 3D spheroids after the treatment were analyzed by flow cytometry (Figure 3C). This figure was created from the PI (fluorescence intensity) data, wherein red and green PI [annexin V-fluorescein isothiocyanate (FITC)] were placed on the *y* and *x* axis, respectively. These results indicate that upon exposure to **1**, there are different cell-death mechanistic pathways for each type of cancer cell. For A549 spheroids, mainly apoptotic and late apoptotic cells are observed (ca. 80 and 10%, respectively). In the case of PANC-1 spheroids, there is a growth in necrosis (ca. 30%) and late apoptosis (ca. 60%). These data may suggest that after the treatment with **1**, there is a quick transformation from primary to late apoptotic cells, along with permeabilization of the plasma membrane in these cell types.

### 2.5 Origin of Cytotoxicity

**Figure 4:**
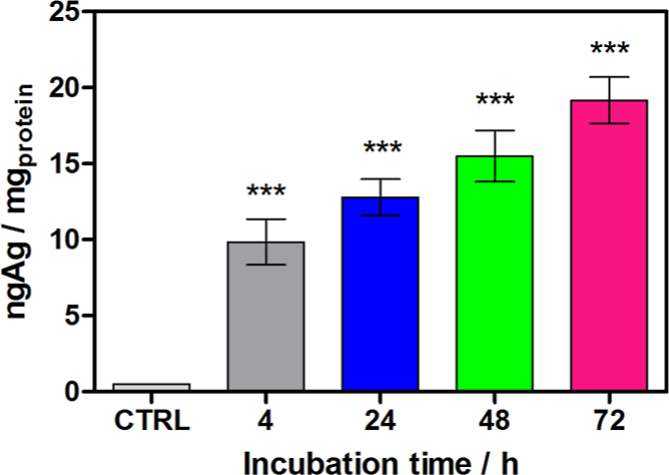
Intracellular concentration of silver (ng Ag per mg of proteins) after incubating PANC-1 with **1** at IC_50_ for 4–72 h. Concentration of Ag for untreated cells has been omitted because it was less than 0.002 ng Ag per mg of proteins. Data are given as mean ± SEM (*** represents *P*-value < 0.001, ** *P*-value < 0.01, and * *P*-value < 0.5).

**Figure 5:**
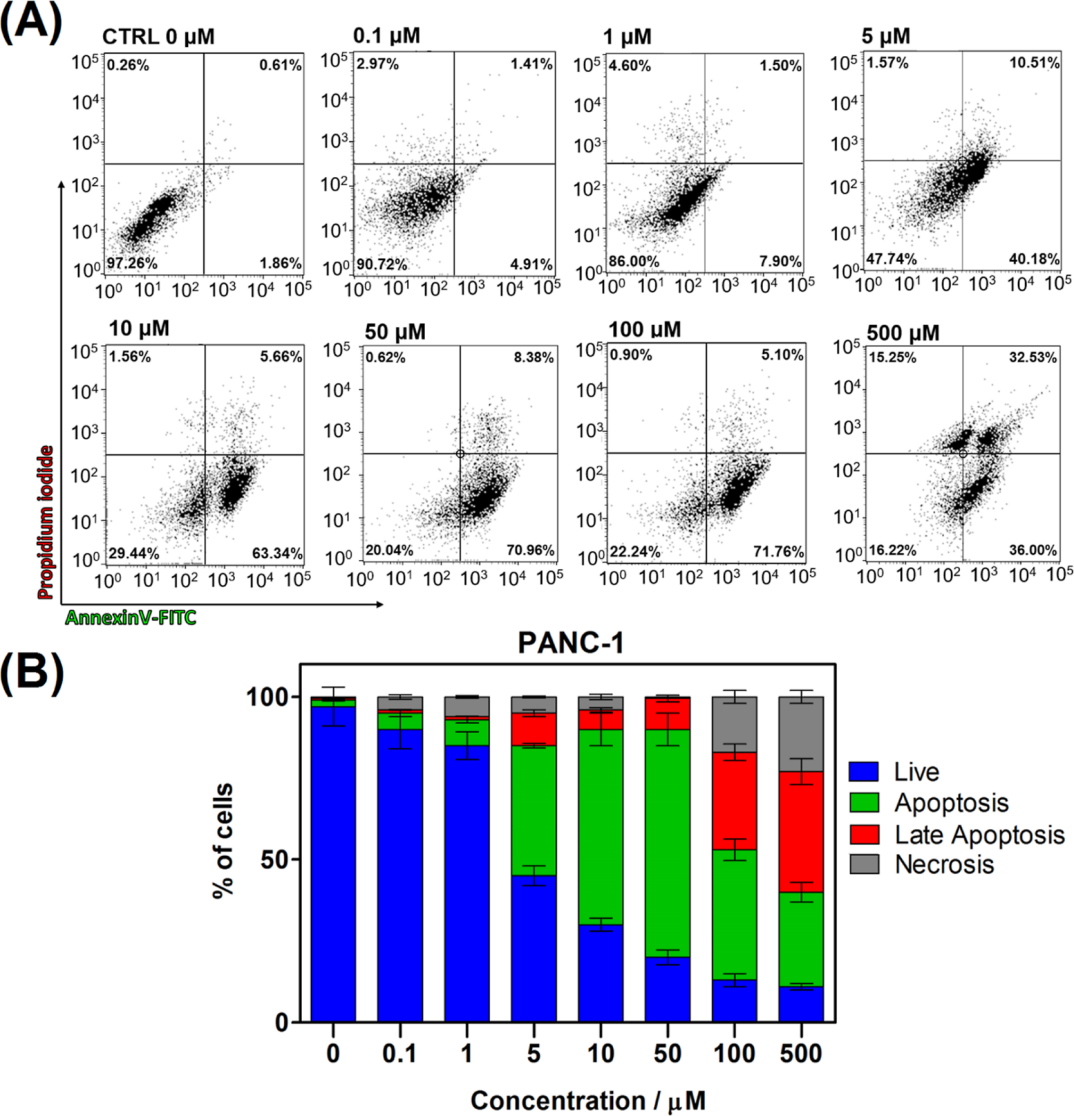
Flow cytometry analysis of dead and viable cells by staining with PI/annexin V–FITC. (A) Representative plots for PANC-1 cells following 24 h treatment with increased concentration of **1**. (B) Percentage dependence of viable cells (annexin V-(−)), necrosis (annexin V(−) and PI(+)), early apoptosis (annexin V(+) and PI(−)), and late apoptosis (annexin V(+) and PI(−)). Data refer to mean ± SD on the basis of three tests. Table S2 contains additional data on statistical significance (Supporting Information).

To correlate the cytotoxicity of **1** with cellular uptake, a silver content in PANC-1 cells after 4, 24, 48, and 72 h treatment with this CP at IC_50_ concentration was evaluated by inductively coupled plasma mass spectrometry (Figure 4). A prolonged incubation time causes an increased accumulation of **1** inside the cells. Then, the determination of the type of cellular death was performed by flow cytometry, allowing the establishment of an accidental (necrosis) or programmed (apoptosis) cell death. After incubating **1** with PANC-1 for 24 h, double staining of cells was performed with PI and the annexin V–FITC conjugate. Incubation with an increased concentration of **1** leads to a significantly augmented population of annexin V-(+) cells. This clearly indicates that an early phase of apoptosis is the principal type of cell death. Moreover, no indication of the PI (+) cells points out the absence of necrotic cells in the analyzed population (Figure 5).

**Figure 6:**
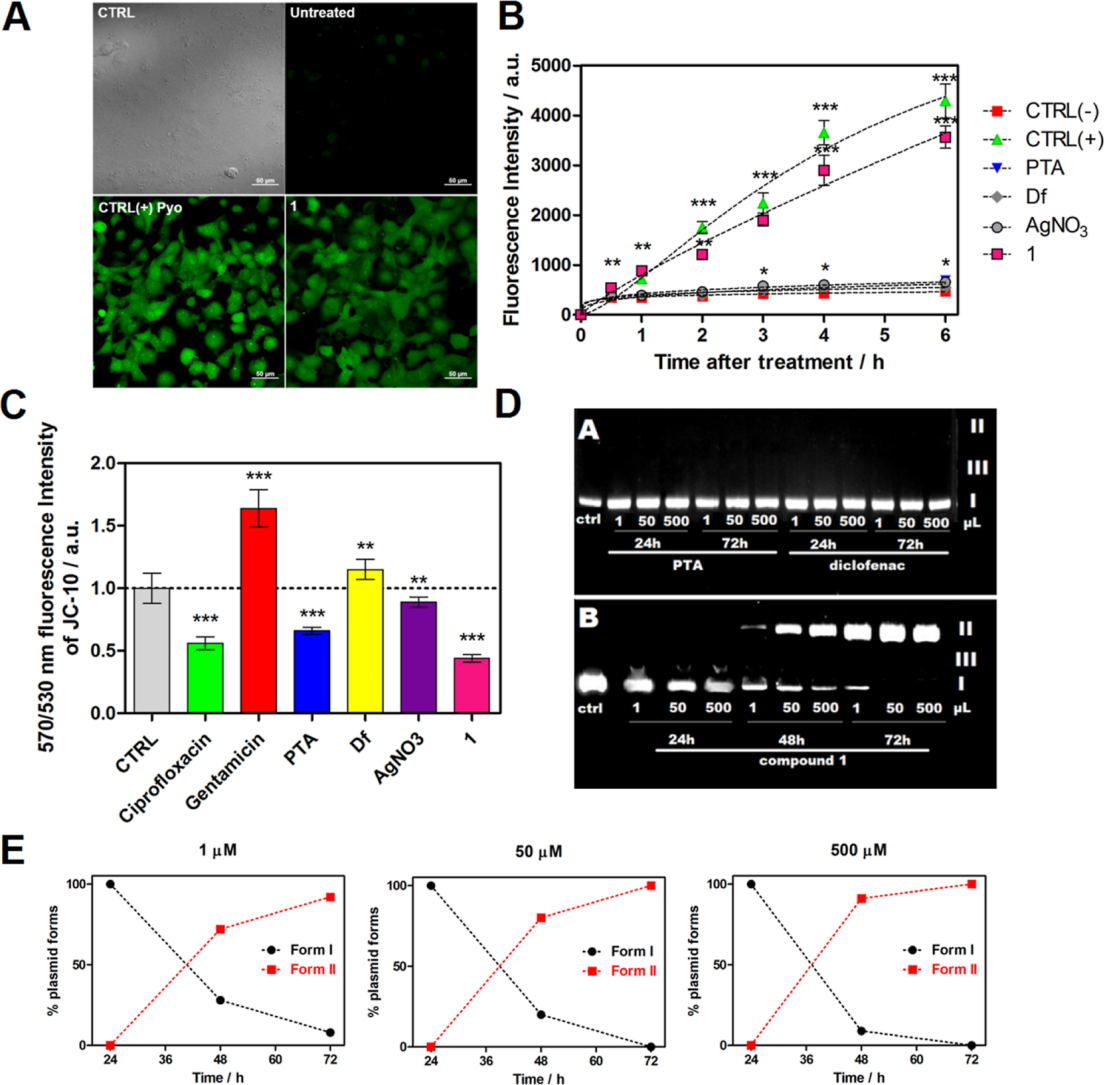
(A) Confocal images of PANC-1 cells treated with **1** and in situ ROS generation after 4 h of incubation. CTRL—untreated control, bright-field image; untreated—cells’ fluorescence image after treatment with **1**; CTRL(+) pyo—cells’ image after treatment with pyocyanin (pyo) during 4 h; positive control and **1**—cells’ image after treatment with **1**, analyzed after 4 h (λ_em_ 524 nm, λ_ex_ 505 nm); scale bar: 50 μm. (B) Level of oxidative stress induced in PANC-1 cells during treatment with **1**, PTA, NaDf, and AgNO_3_ expressed as fluorescence intensity dependence on time (up to 6 h). Pyocyanin- untreated and -treated cells were applied as ROS negative and positive control tests, respectively. (C) Changes of mitochondrial membrane potential by a mitochondrial probe JC-10 for **1**, PTA, NaDf, AgNO_3_, and controls (ciprofloxacin and gentamicin) at respective concentrations based on the molar ratio 1:1:1, incubation time: 4 h. (D) Cleavage of the pBR322 plasmid (agarose gel electrophoresis experiment) in the presence of (A) PTA and NaDf and (B) **1** in dimethylformamide (DMF) (typically 10% DMF solution) at different concentrations (1, 50, and 500 μM) and incubation times (72, 48, and 24 h). (e) Densitometry analysis of plasmid cleavage by **1** (*X* axis: time [h]; *Y* axis: % plasmid forms). Data are given as mean ± SD (*** represents *P*-value < 0.001, ** *P*-value < 0.01, and * *P*-value < 0.5).

To gain further information on the action mode, the influence of **1**, PTA, and NaDf on an intracellular level of ROS (reactive oxygen species) in a selected cancer cell line was evaluated by the Cyto-ID hypoxia/oxidative stress detection assay (Figure 6A,B). In contrast to both ligand precursors (PTA and NaDf) and silver salt, **1** causes a significant time-dependent growth in ROS production in the experiments with the PANC-1 cell line. A resulting fluorescent intensity (a.u.) of **1** is much superior to that of PTA and diclofenac. Intracellular ROS generation represents a key variable for the death of cancer cells realized via the apoptotic pathway as a plenitude of chemotherapeutics induce apoptosis in this way. Thus, an enhanced generation of ROS by the Ag(I) derivative bearing PTA and diclofenac can indicate their significant role in cell death. As an important indicator of mitochondrial dysfunction, the variation in mitochondrial membrane potential was monitored by the JC-10 probe (Figure 6C) aiming to understand the importance of mitochondrial disorder during the cancer cell death induced by **1**.

Both **1** and PTA cause a significant decrease in the 570/530 nm ratio of the fluorescence intensity of the JC-10 probe, in contrast to diclofenac, which shows an increase in mitochondrial potential. Interestingly, this increase caused by **1** is almost double if compared to PTA. This indicates a plausible synergic effect of the different components in **1** (Ag ions, PTA, and anti-inflammatory drug diclofenac), leading to a significantly stronger disorder in the mitochondrial potential activity that could be expected from a mixture of individual components. It is well documented that the disrupted mitochondrial activity is more challenging to restore in cancer cells and these are not able to recover from such severe damage. This conduces to permeabilization of mitochondrial outer membrane and activation of the cell death machinery—apoptosis or autophagy.^38^ Furthermore, since mitochondria may independently generate an elevated ROS level under the cellular stress conditions (e.g., treatment by a metal complex), this may also result in the oxidative damage of mitochondria, subsequently leading to a release of intermembrane space proteins and finally to the death of cells. Hence, the obtained data corroborate that the induction of apoptosis is related to the behavior of mitochondria.

Then, the interaction between DNA and diclofenac, PTA, or **1** was evaluated by the gel electrophoresis of pBR322 plasmid to determine an ability to cause double- and/or single-strand DNA damages (Figure 6D). The double-strand damage may result in the appearance of different forms of DNA (linear form III and relaxed form II), which can be quantified by densitometry analysis (Figure 6E). There is no degradation of DNA caused by PTA and diclofenac during 24 h (Figure 6D,E), even at their high concentration that can exceed the values of IC_50_ (500 μM). Prolongation of the incubation period until 48 h results in the DNA double cleavage at all tested concentrations of **1** (48 h, single cleavage of DNA equals 72% for *C* = 1 μM, 80% for *C* = 50 μM, 91% for *C* = 500 μM). After 72 h of incubation, there is DNA damage (single-strand) with complete disappearance of the superhelical form at 50 and 500 μM concentrations of **1**, while for the 1 μM concentration, only 8% of the superhelical form is left. The linear form of DNA plasmid was not detected in the above experiments. These data indicate that DNA damage depends on the incubation time rather than on the concentration of **1**.

**Figure 7:**
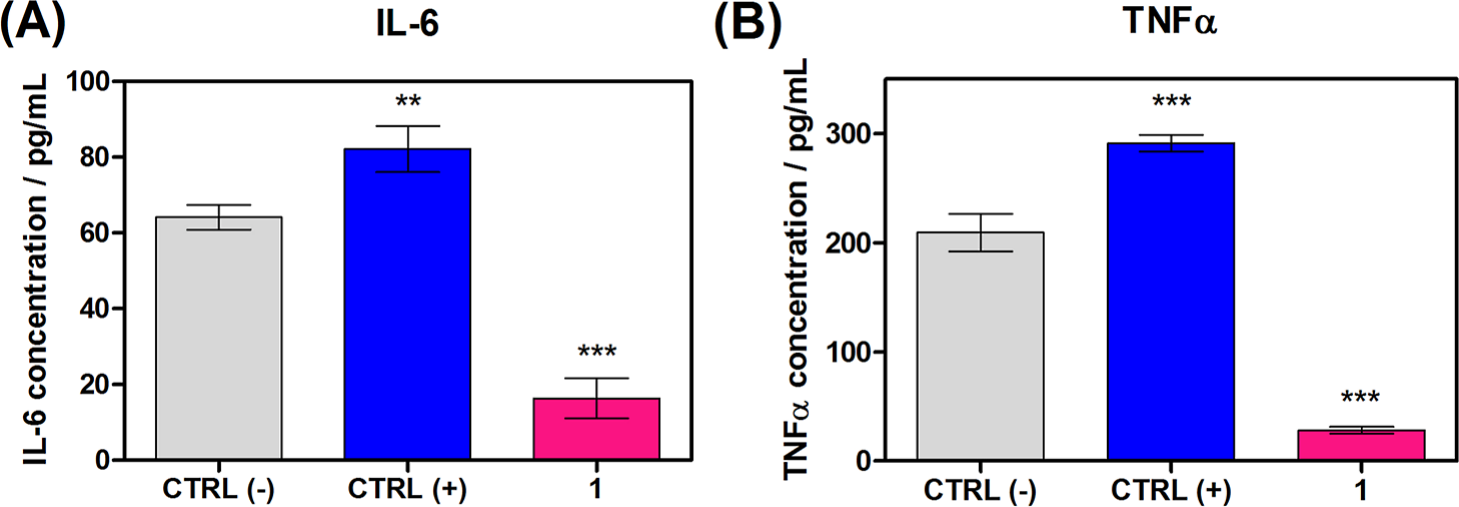
(a) IL-6 and (b) TNF-α production measured after incubation with **1** (*c* = 1 μM), ctr(−) supernatant of untreated cells, ctrl(+) IL6, and TFN-*a*, respectively. Data are represented as mean ± SD (*** represents *P*-value < 0.001, ** *P*-value < 0.01, and * *P*-value < 0.5).

Finally, to confirm an anti-inflammatory activity of **1**, we selected TNF-α (and tumor necrosis factor-alpha) and IL-6 (cytokine interleukin-6) for further study. These are significant factors in physiological neural tissue homeostasis and inflammatory disorder pathogenesis.^43,44^ After incubating PANC-1 cells with and without compound **1**, the activities of TNF-α and IL-6 in the cellular medium were established (Figure 7). As a result of cells’ incubation with **1**, there is a decrease of IL-6 from 60 to 18 pg/mL, as well as a reduction of TNF-α from 200 to 40 pg/mL. It is clear that CP **1** inhibits the activities of these two markers (TNF-α and IL-6). All these results point out that diclofenac does not lose its intrinsic anti-inflammatory properties after coordination with Ag(I).

## 3 Conclusions

In summary, a unique bioactive Ag(I)/PTA/diclofenac CP was prepared and fully characterized, and its therapeutic potential in targeted anticancer therapy was evaluated. Hybrid materials based on Ag(I) derivatives recently gained remarkable importance due to their significant activity toward tumor cells along with much lower severe toxicity against normal cells. The innovative approach proposed herein is based on an additional introduction of the NSAID (diclofenac) to the silver(I)-organic network driven by the water-soluble aminophosphine PTA. As a result, the obtained CP displays a highly probable synergic bioactivity effect of its different components and is capable of performing at least two therapeutic functions—anticancer and demulcent activity. Such a multifunctional metallodrug (CP **1**) thus offers a novel chemotherapeutic approach that can maximize the therapeutic efficacy and minimize systemic toxicity. Notably, **1** exhibits a very significant cytotoxic action against different lines of cancer cells with the IC_50_ parameters as low as 3.1 μM (PANC-1) and very remarkable SI, reaching 28.1 for PANC-1 with respect to normal MRC5 cells. Additionally, the 3D model representing human pancreas/duct carcinoma (PANC-1) and human lung adenocarcinoma (A549) was designed and applied as a clear proof of the remarkable therapeutic potential of **1**. The obtained experimental data indicate that **1** induces an apoptotic pathway via ROS generation, targeting mitochondria due to their membrane depolarization. The preliminary in vitro results are extremely promising, also if compared to a reference cisplatin drug, and encourage further research on explaining the observed mode of action. Compound **1** is thus a unique silver(I) CP with significant simultaneous cytotoxic and anti-inflammatory activity. This compound also widens a family of bioactive silver CPs^45,46^ and, in particular, metal-PTA derivatives with therapeutic potential,^47^ showing that such water-soluble aminophosphine is a promising P,N-linker for generating bioactive coordination networks incorporating drugs as ligands.

## 4 Experimental Section

### 4.1 Materials and Analytical Methods

All chemicals were obtained from commercial suppliers, with an exception of 1,3,5-triaza-7-phosphaadamantane (PTA) that was prepared via an established protocol.^48,49^ Elemental (C/H/N) analysis data were obtained on a VarioELCube Elemental Analyser (University of Wrocław, Faculty of Chemistry, Laboratory of Elemental Analysis). Infrared spectra (FTIR) were recorded on Bio-Rad FTS 3000MX or Bruker IFS 1113v or instruments in the 4000–400 cm^–1^ interval (University of Wrocław, Faculty of Chemistry, Laboratory of Infrared Spectroscopy); abbreviations: br., broad; w, weak; m, medium; s, strong; vs, very strong. ^1^H, ^13^C{^1^H}, and ^31^P{^1^H} NMR spectroscopy measurements were performed on a Bruker 500 AMX spectrometer at room temperature (abbreviations: br., broad, t, triplet; d, doublet; s, singlet). Chemical shift values (δ) are given in ppp relatively to Si(Me)_4_ (^1^H and ^13^C spectra) or external H_3_PO_4_ (aq. 85%, ^31^P spectra). The H_2_O/CH_3_OH solutions (10^–3^ M) of **1** were used for ESI-MS(±) experiments that were carried out on a Bruker MicroOTOF-Q mass spectrometer with an ESI source.

### 4.2 Synthesis of [Ag(μ-PTA)(Df)(H_2_O)]_*n*_·3*n*H_2_O (**1**)

#### 4.2.1 Solution Self-Assembly Synthesis

AgNO_3_ (17 mg, 0.1 mmol), diclofenac sodium salt (NaDf, 31.8 mg, 0.1 mmol), and PTA (15.8 mg, 0.1 mmol) were combined with a H_2_O/MeOH solution (5 + 5 mL). The resulting mixture was left stirring at ambient temperature in air for 1 h and then adjusted to pH = 8 by adding 1 M solution of NH_3_OH (aq). The resulting transparent solution was filtered off and kept undisturbed to allow crystallization at ambient temperature. The light-gray or colorless microcrystals of **1** were obtained in a 40% yield (relatively to AgNO_3_). Anal. Calcd for C_20_H_24_Cl_2_AgN_4_O_3_P (**1**–3H_2_O, MW 578.2): C, 41.55; N, 9.69; H, 4.18. Found: C, 41.65; N, 9.62; H, 4.18. IR (KBr, cm^–1^): 3438 (s, br.): ν(H_2_O), 3232 (m) (NH), 3057 (w), 2964 (w) ν_as_(CH), 2941 (w) ν_s_(CH), 1586 (vs) ν_as_(COO), 1560 (w), 1505 (s) and 1450 (s) ν_s_(COO), 1417 (w), 1362 (s), 1294 (m), 1277 (w), 1241 (m), 1190 (w), 1178 (w), 1153 (w), 1104 (m), 1039 (w), 1017 (vs), 971 (vs), 950 (vs), 899 (w), 890 (w), 861 (w), 821 (w), 775 (m), 743 (vs) (C–Cl), 715 (w), 671 (w), 643 (w), 597 (m), 580 (m), 565 (m), 543 (w), 528 (w) 496 (w), 467 (w), 456 (w). ^1^H NMR (D_2_O, 500.1 MHz): δ 7.49 (d, 8.2 Hz, 2H, C_6_H_3_, Df), 7.26 (dd, 7.25 and 1.5 Hz, 1H, C_6_H_4_, Df), 7.15 (t, 8.2 Hz, 1H, C_6_H_3_, Df), 7.12 (dt, 8.0 and 1.5 Hz, 1H, C_6_H_4_, Df), 6.97 (dt, 7.25 and 1.1 Hz, 1 H, C_6_H_4_, Df), 6.49 (d, br, 8.0 Hz, 1H, C_6_H_4_, Df), 4.63 and 4.52 (*J*_AB_ = 13.0 Hz, 6H, NCH_A_H_B_N, PTA), 4.27 (s, br, 6H, PCH_2_N, PTA), 3.66 (s, CH_2_COO, 2H, Df). ^1^H NMR (DMSO-*d*_6_, 500.1 MHz): δ 9.27 (br s, 1H, NH, Df), 7.48 (d, 8.0 Hz, 2H, C_6_H_3_, Df), 7.10 (t, 8.0 Hz, 1H, C_6_H_3_, Df), 7.09 (d, 7.6 Hz, 1H, C_6_H_4_, Df), 6.97 (dt, 7.6 Hz and 1.5 Hz, 1H, C_6_H_4_, Df), 6.78 (dt, 7.25 and 1.1 Hz, 1H, C_6_H_4_, Df), 6.27 (d, 7.25 Hz, 1H, C_6_H_4_, Df), 4.54 and 4.39 (*J*_AB_ = 12.5 Hz, 6H, NCH_A_H_B_N, PTA), 4.23 (d, ^2^*J*_P–H_ = 2.7 Hz, 6H, PCH_2_N, PTA), 3.48 (s, CH_2_COO, 2H, Df). ^13^C[^1^H] NMR (DMSO-*d*_6_, 125.8 MHz): 176.2 (s, COO, Df); 142.9, 137.7, 130.3, 129.2, 128.9, 127.2, 126.3, 124.4, 120.3, 115.8 (10s, C_6_H_3_, C_6_H_4_, Df); 72.1 (d, ^3^*J*_P–C_ = 6.4 Hz, NCH_2_N, PTA); 50.1 (d, ^1^*J*_P–C_ = 7.3 Hz, PCH_2_N, PTA); 42.3 (s, CH_2_COO, Df). ^31^P[^1^H] NMR (DMSO-*d*_6_, 202.5 MHz): −83.6 (s, PTA). ESI-MS(±) (CH_3_OH/H_2_O), MS(+) *m*/*z*: 422.1 (60%) [Ag(PTA)_2_]^+^, 558.9 (50%) [Ag(PTAH)(Df)]^+^, 823.9 (100%) [Ag_2_(PTA)_2_(Df)]^+^; MS(−) *m*/*z*: 613 (30%) [Ag(PTA)(Df)(H_2_O)_3_ – H]^−^. TG–DTA for **1** (10 °C/min, N_2_ flow): 20–100 °C [−3(H_2_O)], Δ*m*: % 6.8 expt., 8.5 calcd.) >200 °C (dec). Purity of **1** is >95% according to PXRD data.

#### 4.2.2 Mechanochemical LAG

AgNO_3_ (17 mg, 0.1 mmol), sodium diclofenac (NaDf, 31.8 mg, 0.1 mmol), and PTA (15.8 mg, 0.1 mmol) were ground manually for 1 h using a pestle and mortar in the presence of one drop of a CH_3_CN/H_2_O mixture (1/1). The obtained white powder was collected and washed with H_2_O, CH_3_OH, and Et_2_O (3 × 0.5 mL). Yield: 90% relatively to AgNO_3_. Anal. Calcd for C_20_H_24_Cl_2_AgN_4_O_3_P (**1**–3H_2_O, MW 578.2): C, 41.55; N, 9.69; H, 4.18. Found: C, 41.34; N, 9.50; H, 4.43. IR (KBr, cm^–1^): 3444 (s, br.) ν(H_2_O), 3231 (m) (NH), 3057 (w), 2964 (w) ν_as_(CH), 2941 (w) ν_s_(CH), 1586 (vs) ν_as_(COO), 1560 (w), 1505 (s) and 1450 (s) ν_s_(COO), 1417 (w), 1411 (w), 1361 (s), 1294 (m), 1277 (w), 1241 (m), 1189 (w), 1178 (w), 1153 (w), 1105 (m), 1039 (w), 1017 (vs), 971 (vs), 950 (vs), 899 (w), 890 (w), 861 (w), 821 (w), 784, (m), 743 (vs) (C–Cl), 715 (w), 671 (w), 644 (w), 598 (m), 580 (m), 568 (w), 543 (w), 528 (w) 496 (w), 467 (w), 456 (w). NMR, IR, and ESI-MS data are essentially similar to the spectra of **1** that were obtained by the solution self-assembly method.

### 4.3 X-ray Crystallography

Crystal data for **1**: C_20_H_30_AgCl_2_N_4_O_6_P, *M* = 632.22, *a* = 14.8109(5) Å, *b* = 14.6858(4) Å, *c* = 11.4625(3) Å, β = 97.142(3)°, *V* = 2473.86(13) Å^3^, *T* = 293(2) K, space group *P*2_1_/*c*, *Z* = 4, Mo Kα, 21052 reflections measured, 4363 independent reflections (*R*_int_ = 0.0735), *R*_1_ = 0.0462 (*I* > 2σ(I)), w*R*(*F*^2^) = 0.1260, GoF (*F*^2^) = 1.013. Single-crystal data collection was carried out on an Xcalibur (Oxford Diffraction) diffractometer using ω-scan and a graphite-monochromated Mo K (λ = 0.71073 Å) radiation. The diffractometer was equipped with a Sapphire2 CCD detector and an Oxford Cryosystems open-flow nitrogen cryostat. CrysAlis PRO (Rigaku Oxford Diffraction, Wrocław, Poland) software was used for cell refinement, data reduction, analysis, and absorption correction.^50^ The crystal structure of **1** was determined by direct methods (SHELXT-2014/5), and the refinement was made by the full-matrix least-square technique on *F*^2^ (SHELXL-2018/3).^51,52^ The H4 atom from the amino group was localized in the Fourier map difference and freely refined. Hydrogen atoms of H_2_O moieties O1W–O3W were localized and refined with restrained O–H distances (for all water molecules) and H–O–H angle (for O3W). The remaining H atoms were added to the calculated sites and refined using the *U*_iso_ = 1.2*U*_eq_ model. Topos software was used for structural visualization.^53^

PXRD was performed on a Bruker D8 ADVANCE diffractometer (Cu Kα radiation). The experimental powder data were processed with DiffractWD software.^54^ The theoretical powder X-ray diffractograms were generated with Mercury 2020.1 software.^55^ CCDC 2024943.

## Supporting Information Available

Additional experimental procedures, structural representations; IR, NMR, and ESI-MS spectra; PXRD and thermogravimetric analysis plots; and additional results (PDF)

## Abbreviations

A549: lung adenocarcinoma
annexin V–FITC: annexin V–fluorescein isothiocyanate conjugate
AO/PI: acridine orange/propidium orange
API: active pharmaceutical ingredients
ATCC: American Type Culture Collection
CP: coordination polymer
Df^–^: diclofenac anion
DMEM: Dulbecco’s modified Eagle’s medium
DU-145: prostate carcinoma
EDTA: ethylenediaminetetraacetic acid tetrasodium salt
FBS: fetal bovine serum
HaCat: keratinocytes
IC_50_: half-maximal inhibitory concentration
IL-6: interleukin-6
TNF-α: tumor necrosis factor-alpha
JC-10: cationic dye, commonly used to determine mitochondrial membrane potential
LAG: liquid-assisted grinding
MCF-7: breast adenocarcinoma
MRC5: Medical Research Council cell strain 5
MTT: 3-(4,5-dimethylthiazol-2-yl)-2,5-diphenyltetrazolium bromide
MEM: modified Eagle medium
MMP: mitochondrial membrane potential
NaDf: sodium diclofenac salt
PANC-1: pancreas/duct carcinoma
pBR322: one of the most commonly used *E. coli* cloning vectors
PBS: phosphate-buffered saline
PDAC: pancreatic ductal adenocarcinoma
PTA: 1,3,5-triaza-phosphaadamantane
S.D.: standard deviation
TG–DTA: thermogravimetry–differential thermal analysis
TME: tumor microenvironment
