# Supplementary material for: Therapeutic Potential of a Water-Soluble Silver-Diclofenac Coordination Polymer on 3D Pancreatic Cancer Spheroids
Source: J Med Chem. 2022 Aug 15;65(16):11100–10. doi: 10.1021/acs.jmedchem.2c00535 (PMC9776540; doi:10.1021/acs.jmedchem.2c00535)
Supplement: Supplementary file 1 — jm2c00535_si_001.pdf [file jm2c00535_si_001.pdf]

Supporting Information

Therapeutic Potential of a Water-Soluble Silver-Diclofenac Coordination Polymer on 3D Pancreatic Cancer Spheroids

Sabina W. Jaros,[§] Urszula K. Komarnicka,[§] Agnieszka Kyzioł,[#] Barbara Pucelik,[†] Dmytro S. Nesterov,[‡] Alexander M. Kirillov[‡,*] and Piotr Smoleński[§,*]

[§]Faculty of Chemistry, University of Wrocław, F. Joliot-Curie 14, 50-383, Wrocław, Poland; E-mail: piotr.smolenski@chem.uni.wroc.pl

[#]Faculty of Chemistry, Jagiellonian University, Gronostajowa 2, 30-387 Kraków, Poland

[†]Malopolska Centre of Biotechnology, Jagiellonian University, Gronostajowa 2, 30-387 Kraków, Poland

[‡]Centro de Química Estrutural, Institute of Molecular Sciences, Departamento de Engenharia Química, Instituto Superior Técnico, Universidade de Lisboa, Av. Rovisco Pais, 1049-001 Lisbon, Portugal; E-mail: kirillov@tecnico.ulisboa.pt

Table of Contents

Experimental Procedures

Cell Cultures S-3
Cytotoxic Activity S-3
Acridine Orange (OA) and Propidium Iodide PI Staining S-3
Assessment of Cytotoxic Effect in the 3D Culture Model S-4
Silver Uptake S-4
Cell Death Analysis by Flow Cytometry S-5
Fluorescence Microscopy and Oxidative Stress Induction in Vitro S-5
Detection of Mitochondrial Membrane Potential (Δψ) S-5
DNA Strand Break Analysis S-5
Inflammatory Properties S-6
Stability tests for **1** S-6
Octanol-Water Partition Coefficient determination S-6

Supplementary Figures

S1. PXRD analysis of **1** S-7
S2. 2D H-bonded water layer in **1** and its topological representation S-7
S3. FT-IR spectrum of **1** S-8
S4. Fragment of the ESI-MS(+) plot of **1**. S-8
S5. Experimental and simulated isotopic distribution patterns for selected fragments in ESI-MS(+) of **1**. S-9
S6. Fragment of the ESI-MS(−) plot of **1**. S-9
S7. Experimental and simulated isotopic distribution pattern for $[Ag(PTA)(Df)(H_2O)_3 - H]^-$ ESI-MS(−) of **1.** S-10
S8. ^1H NMR spectrum of **1** in D_2O S-10
S9. ^1H NMR spectrum of **1** in DMSO-d_6 S-10
S10. $^{31}P[^1H]$ NMR spectrum of **1** in DMSO-d_6 S-11
S11. $^{13}C[^1H]$ NMR spectrum of **1** in DMSO-d_6 S-11
S12. ^1H NMR spectrum of **1** in DMSO-d_6/D_2O = 5/2 S-11
S13. $^{31}P[^1H]$ NMR spectrum of **1** in DMSO-d_6/D_2O = 5/2 S-12

S14. ^{1}H NMR spectrum of **1** in DMSO-d_6/D_2O = 5/2 in presence of NaCl (c=5 mM) S-12
S15. $^{31}P[^{1}H]$ NMR spectrum of **1** in DMSO-d_6/D_2O = 5/2 in presence of NaCl (c=5 mM) S-12
S16. ^{1}H NMR spectrum of **1** in DMSO-d_6/D_2O = 5/2 in presence of NaCl (c=50 mM) S-13
S17. $^{31}P[^{1}H]$ NMR spectrum of **1** in DMSO-d_6/D_2O = 5/2 in presence of NaCl (c=50 mM) S-13
S18. ^{1}H NMR spectrum of **1** in DMSO-d_6/D_2O = 5/2 in presence of acetic acid buffer (pH=5.5) S-13
S19. $^{31}P[^{1}H]$ NMR spectrum of **1** in DMSO-d_6/D_2O = 5/2 in presence of acetic acid buffer (pH=5.5) S-14
S20. ^{1}H NMR spectrum of **1** in DMSO-d_6/D_2O = 5/2 in presence of acetic acid buffer (pH=4.0) S-14
S21. $^{31}P[^{1}H]$ NMR spectrum of **1** in DMSO-d_6/D_2O = 5/2 in presence of acetic acid buffer (pH=4.0) S-14
S22. TGA analysis of **1** S-15
S23. Positive ESI-MS^2 plot of cation $[Ag(PTA)_2]^+$ fragment of **1** S-15
S24. Positive ESI-MS^3 plot of cation $[Ag(PTA)_2]^+$ fragment of **1** S-16

Supplementary Tables

S1. Results of NMR tests in presence of NaCl and acetic acid buffer S-17
S2. Statistical significance of percentage dependence of viable cells for Fig. 5 S-17
S3. Surviving fractions (%) estimated relative to untreated controls of selected cells. S-18

References S-19

Experimental Procedures

Cell Cultures

MCF7 cell line (human breast adenocarcinoma, morphology: epithelial-like, ATCC: HTB-22), A549 cell line (human lung adenocarcinoma, morphology: epithelial, ATCC: CCL-185), PANC-1 cell line (human pancreatic/duct carcinoma, morphology: epithelial, ATCC: CRL-1469) and HaCat cell line (human keratinocyte, morphology: epidermal, CSL Cell Lines Service GmbH); were cultured in Dulbecco's Modified Eagle's Medium (DMEM, Corning) with phenol red, supplemented with 10% fetal bovine serum (FBS) and with 1% streptomycin/penicillin. DU-145 cell line (human prostate carcinoma, derived from metastatic site: brain, ATCC: HTB-81) and MCR-5 cell line (primary line of human pulmonary fibroblasts, ATCC: CCL-171) were cultured in minimum essential medium (MEM, Corning) with only 10% fetal bovine serum (FBS). Cultures were incubated at 37 °C under a humidified atmosphere containing 5% CO_2. Cells were transferred using a solution containing 0.05% trypsin and 0.5 mM EDTA. All media and other ingredients were purchased from ALAB, Poland.

Cytotoxic Activity

Cytotoxicity was assessed by MTT assay performed according the protocols described elsewhere.[S1] In brief, 1×10^4 cells per well, seeded in 96-well flat bottom microtiter plate, were incubated with the tested compounds (in the appropriate media) at various range of concentrations for 24 h. After that time, solutions of compounds were washed out, cells were washed three times with PBS and fresh medium was applied. After next 72 h cell viability was determined using the Hill equation (Origin 9.0) with regard to the untreated cells (control), where y_0 – untreated cell control (which was set to 100% viability), y_{100} – a lysis control (where the cells were treated with 0.5% triton X-100 was set to 0% viability, which was found to be sufficient to induce 100% cell death), IC_{50} – values for the concentration [c] at which the viability of the cells reaches 50%, H – the Hill coefficient (describing cooperativity):

$$y = \mathrm{y}_0 + \frac{(\mathrm{y}_{100} - \mathrm{y}_0 b)[c]^H}{(\mathrm{IC}_{50})^{\mathrm{H}} + [c]^H}$$

Each compound concentration was tested in five replicates and repeated at least three times. Determined values of IC_{50} are given as mean + S.D. (Standard Deviation).

Cisplatin was tested as a reference anticancer drug. Furthermore, the cytotoxic effect of $AgNO_3$ was evaluated to exclude a significant influence of free Ag(I) ions to overall observed cytotoxicity. Up to a concentration of 100 µM, silver(I) nitrate does not cause significant toxic effects in our *in vitro* experimental system and there is no any visible precipitation.

Acridine Orange (OA) and Propidium Iodide PI Staining

AO is a nucleic acid-binding fluorophore that is cell membrane permeable and suitable for selective staining of nucleated living cells. PI is a nucleic acid-binding dye that is impermeable to live cells and suitable for staining dead or dying nucleated cells. All live, nucleated cells fluoresce green due to AO, and dead, nucleated cells are stained with both AO and PI and fluoresce red. For analysis of **1**-induced cytotoxicity, 30×10^3 of PANC-1 cells were seeded in 12-well plates on microscopic slides and incubated at 37 °C for 24 h. Then, cells were washed with PBS and incubated with compound **1**, PTA, NaDf (diclofenac), and $AgNO_3$ at the respective concentrations based on the molar ratio 1:1:1:1 for 24 h.

After this incubation, cells were washed two times with PBS and (100 µg/mL) and PI (10 µg/mL) was added to each well (500 µL). After 5 min later, the cells were visualized via a confocal fluorescence microscope Zeiss880 (Carl Zeiss, Germany) with a 40 × objective. Images were analyzed by Zeiss ZEN Software.

Assessment of Cytotoxic Effect in the 3D Culture Model

3D culture: A549 or PANC-1 cells were grown in DMEM (high glucose) supplemented with 10% heat-inactivated fetal bovine serum and 1% antibiotics. For 3D cell culture, the GelTrex matrix was used as a basement membrane, which has an appropriate gel structure and established biological activity to promote the growth and differentiation of various cells. To prepare 3D cultures by the hanging-drop method, 50 µL volumes of cells in the single-cell suspension of 50,000 cells/mL were used. When the cells formed spheroids, they were plated on the GelTrex matrix and were allowed to adhere before adding a complete growth medium with 2% GelTrex. All cultures were maintained in an incubator at 37 °C in an atmosphere of 5% CO_2.

Cytototoxicity assessment with confocal fluorescence microscopy: To visualize the cytotoxicity of **1** against A549 and PANC-1 spheroids, the fluorescence live/dead staining with Hoechst 33342, Calcein AM and PI (propidium iodide) was performed. After the treatment with **1**, media was removed from culture dishes of 3D spheroids by gentle aspiration, followed by a wash with PBS with Ca^{2+} and Mg^{2+} and the incubation at 37 °C for 40 min with 2 µM calcein AM, 10 µg/mL Hoechst 33342, and 2 µM propidium iodide diluted in PBS before imaging. The addition of reagents to each culture dish was staggered such that the incubation period was exactly 40 min for each dish. Then spheroids were washed with PBS and imaged with Olympus BX51 Microscope (Olympus, Tokio, Japan). Digital images were acquired by sequentially rotating filter cubes at each of four spatial fields imaged in each culture dish: a bright-field image, a Hoechst 33342 emission image (exc./em. 361/497), a calcein AM fluorescence emission image (exc./em. 494/517 nm), and propidium iodide with exc./em. 535/517 nm, respectively.

Cell death analysis in 3D spheroids: In order to perform the flow cytometry analysis on spheroids treated with **1** (24 h), the spheroids were dissociated into single cells using trypsin–EDTA 0.25% for 5 min with gentle pipetting up and down to minimize aggregated cell population for the following analysis. The single-cell suspension from the dissociated spheroids was then incubated with Annexin V-FITC Apoptosis Detection Kit (Sigma Aldrich) accordingly to the manufacturer's instruction. The stained cells were analyzed by a flow cytometer (BDAccuri, BD Bioscience) measuring green fluorescence emission for FITC and red fluorescence emission for PI.

Silver Uptake

PANC-1 cells at density of $2×10^6$ cells/2 mL were seeded on 6-well plates and incubated with **1** (c=1 µM for 4 or 24h) at standard conditions (37 °C, 5% CO_2). Solution of the studied compound was removed; the cells were washed twice with PBS buffer and trypsinized. For ICP-MS analysis, the cells were mineralized in 1 mL of 65% HNO_3. Measurement of the concentration of silver ions was determined by a mass spectrometer (ELAN 6100 Perkin Elmer) with an inductively coupled plasma (ICP-MS). Protein content was assessed with Bradford Protein Assay (Thermo Scientific™).[S1] The silver content under each condition is expressed as ng Cu/mg protein. The experiment was repeated at least 3 times and results are presented as mean value + S.D.

Cell Death Analysis by Flow Cytometry

Annexin V Apoptosis Detection Kit FITC (Sigma Aldrich) and Propidium Iodide (Thermo Fischer Scientific, Waltham, Massachusetts, USA) were used to distinguish cell death (apoptotic and necrotic cells) induced by **1** compound quantitatively. For this purpose, complex 1 prepared in a broad range of concertation ranging between 0-500 μM) were incubated for 24 h with PANC-1 cells (seeded at density 5×10^5 cells/mL) in 12-well plates. After this time, the compound solutions were removed, and the cells were washed twice with PBS buffer (phosphate-buffered saline, pH = 7.4). Trypsin was added to the cells and then they were left for 10 min at 37 °C in a humidified atmosphere containing 5% CO_2. The cells were collected, centrifuged, and separated from the supernatant, then washed twice with 0.5 ml PBS buffer and suspended in Binding Buffer. Fifteen minutes before measuring, cells were stained with Annexin V-FITC and PI and incubated in the dark. Viable and dead (early apoptotic, late apoptotic, and necrotic) cells were detected using the BDAccuri flow cytometer (BD Biosciences). The experiment was repeated at least 3 times.

Fluorescence Microscopy and Oxidative Stress Induction in Vitro

Oxidative stress was detected by staining with Cyto-ID Hypoxia/Oxidative Stress Detection Kit for 10 min and examined using a fluorescence confocal microscopy (Zeiss880, Carl Zeiss, Germany). Production of Reactive Oxygen Species (ROS) in PANC-1 cells induced by **1**, PTA, diclofenac and $AgNO_3$ was determined by photometric test – Cyto-ID® Hypoxia/Oxidative Stress Detection Kit according to procedure described elsewhere.[52] Pyocyanin and untreated cells were used as positive and negative controls, respectively. PANC-1 cells (30,000/well) were seeded in the 12-well black-wall plate overnight before the experiments. The following day, cells were washed with HBSS and treated with **1** for various time up to 6 h. After this incubation, cells were washed twice with HBSS and analyzed by fluorescence intensity measurements using Tecan M200 Pro microplate reader (λ_{ex} 505 nm, λ_{em} 524 nm).

Detection of Mitochondrial Membrane Potential (Δψ)

Mitochondrial membrane potential (MMP) depletion was determined by JC-10 Assay (Life Technologies, USA). PANC-1 cells were seeded on 96-well plates at 1×10^4 cells/0.2 mL. After 24h medium was replaced with solutions of **1** at IC_{50} concentration as well as gentamicin (0.5 mg/mL) and ciprofloxacin (10 μg/mL) as positive and negative control, respectively. After that, cells were incubated for 4 h at standard condition (37 °C, 5% CO_2). Then, they were washed twice with PBS buffer and incubated with JC-10 for 1 h. Afterwards, emission was measured at two different excitation wavelengths (λ_{exc} = 540 nm, λ_{em} = 570 nm) and (λ_{exc} = 485 nm, λ_{em} = 530 nm). Results are presented as the intensity ratio of red to green emission (mean + S.D.).

DNA Strand Break Analysis

The ability of **1**, PTA and diclofenac to induce single- or double-strand breaks in plasmid DNA was tested with the pBR322 plasmid (c = 0.5 mg/mL). All compounds were dissolved in water (c = 1 μM-500 μM). After 1 h incubation at 37 °C, the reaction mixtures (20 μL) were mixed with 3 μL of loading buffer (bromophenol blue in 30% glycerol) and loaded on 1% agarose gels, containing EB, in TBE buffer (90 mM Tris–borate, 20 mM EDTA, pH = 8.0). Gel electrophoresis was performed at a constant voltage of 100 V (4 V/cm) for 60 min. The gel was photographed and processed with a Digital Imaging System (Syngen Biotech). For the densitometric analysis the UltraQuant 6.0 program was used.

Inflammatory Properties

IL6 and TFN-α were uantified in the conditioned cell culture medium using the Human IL6 ELISA Kit and TNF alpha Human ELISA Kit, respectively. Study has been performed according to the manufacturer's protocol (Immuniq, Poland - for IL6; Invitrogen, Poland for TFN-α) and using Microplate Readers (Tecan).

4.14. Statistical Analysis. Results are present as mean ± standard deviation (SD) from at least three independent experiments. Statistical significance was determined by one-way or two-way ANOVA with Bonferroni post hoc test using GraphPad Prism version 5.0.0 for Windows, GraphPad Software, San Diego, California USA, www.graphpad.com).

Stability Tests for 1

Compound **1** was air stable at least for one year in the solid state and for minimum one week in DMSO-d_6 with addition of deuterated water and in the light. In a general procedure, **1** was introduced into a NMR tube and dissolved in 0.5 mL of DMSO-d_6 and 0.2 mL of D_2O in air atmosphere. $^{31}P\{^1H\}$ NMR showed that no evident changes were produced in one week at room temperature in air. Effect of pH=4.0 and 5.5 on the stability of **1** was also monitored by NMR spectroscopy, using acetic acid buffer solutions. No significant changes were observed in this pH range (apart from a slight shift of the resonances). Moreover, the influence of pseudophysiological conditions on the stability of **1** was studied over the time intervals (0, 24 and 48 h) and monitored by ^{31}P and ^1H NMR spectroscopy (5 and 50 mM NaCl solutions of DMSO-d_6/D_2O =5/2). Results of these tests are given in Table S1.

Octanol-Water Partition Coefficient Determination

The lipophilicity parameter, log P, values corresponding to the octanol-water partition coefficient were adjusted to the solubility properties of the compounds [Sangster, J.; J. Phys. Chem. Ref. Data, 1989, 1111–1227]. CP **1** was dissolved in water (10^{-4} M) that was previously saturated with octanol. Into a 50 mL flask at 24 ^oC with a magnetic stir bar were introduced initially 10 mL of octanol previously saturated with water and then 10 mL of **1** in water. Two-phase mixture was stirred vigorously for 10 min. and the water phase was measured using the ICP technique. Amount of the Ag concentration was compared with the initial concentration of **1** in water. Value of log P has been found as −0.61.

Supplementary Figures

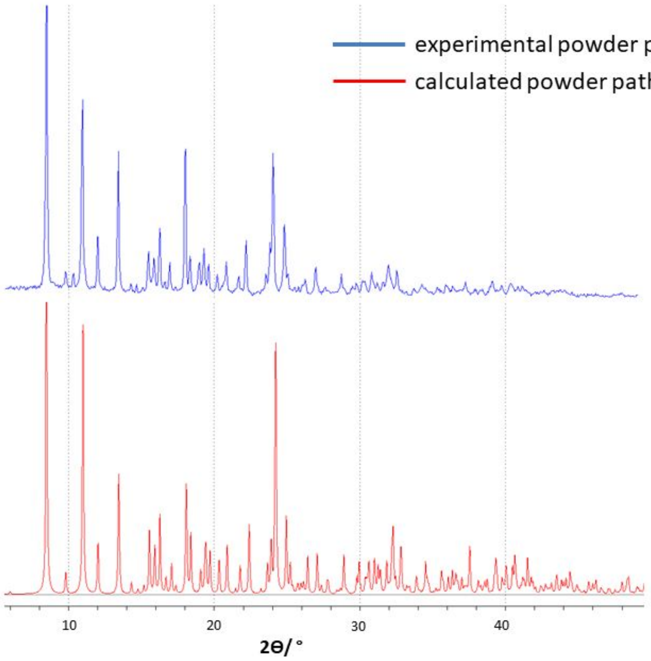

Figure S1. PXRD analysis of **1**, produced by mechanochemical liquid-assisted route in comparison to calculated powder pattern.

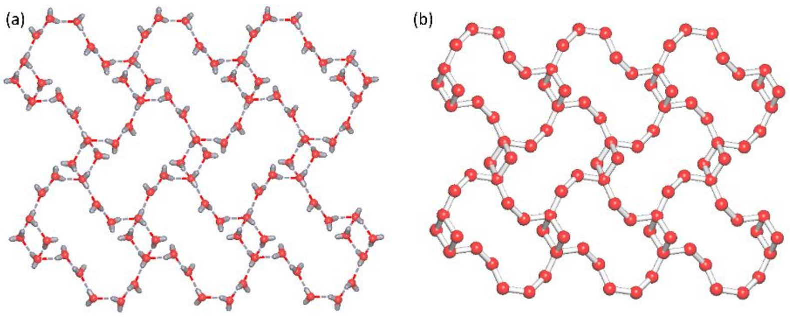

Figure S2. (a) 2D H-bonded water layer in **1** and (b) its topological representation showing a uninodal 3-connected net with the hcb [Shubnikov hexagonal plane net/(6,3)] topology. Views along the *a* axis. (a) O red, H gray. (b) centroids of H_2O molecules (red balls).

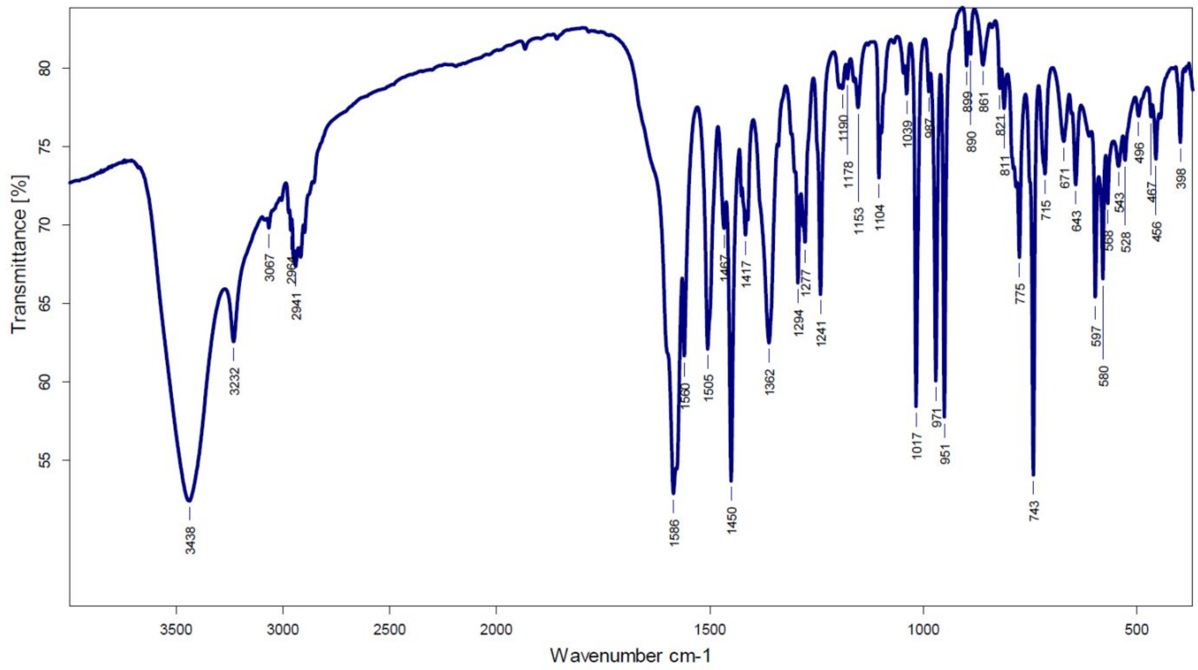

Figure S3. IR spectrum of **1**.

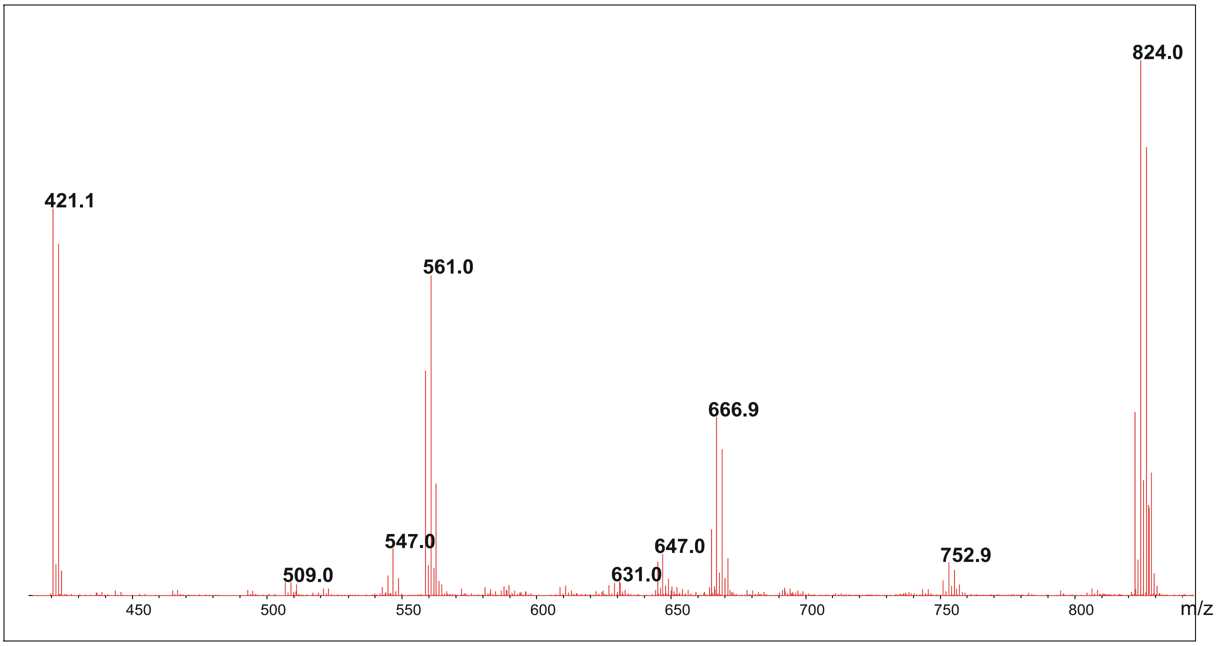

Figure S4. Fragment of the ESI-MS(+) plot of **1**.

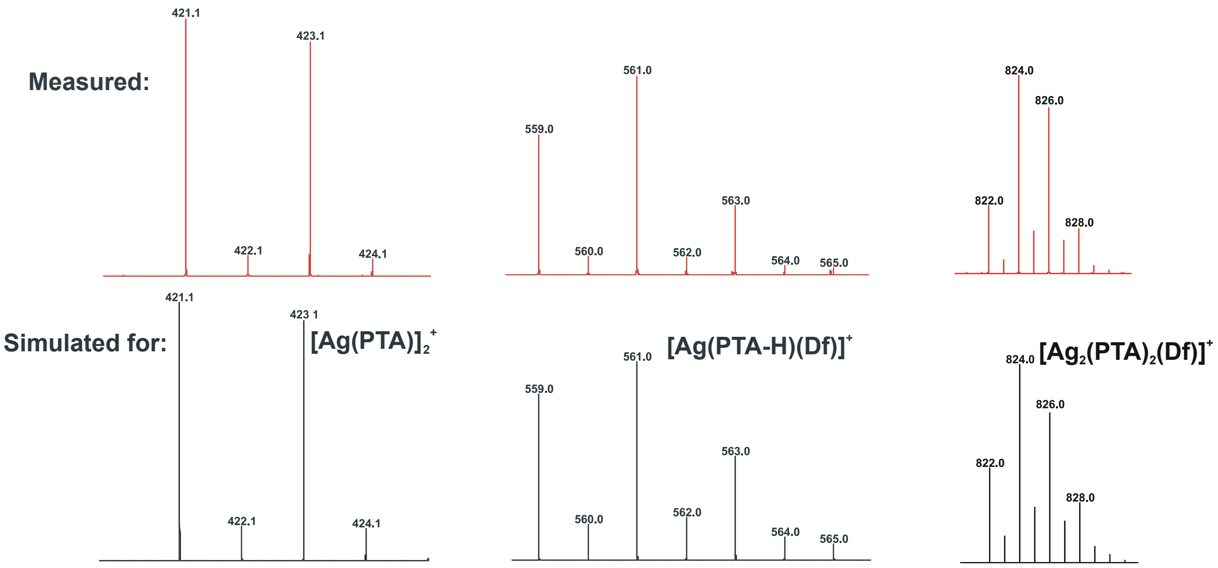

Figure S5. Experimental and simulated isotopic distribution patterns for selected fragments in ESI-MS(+) of **1**.

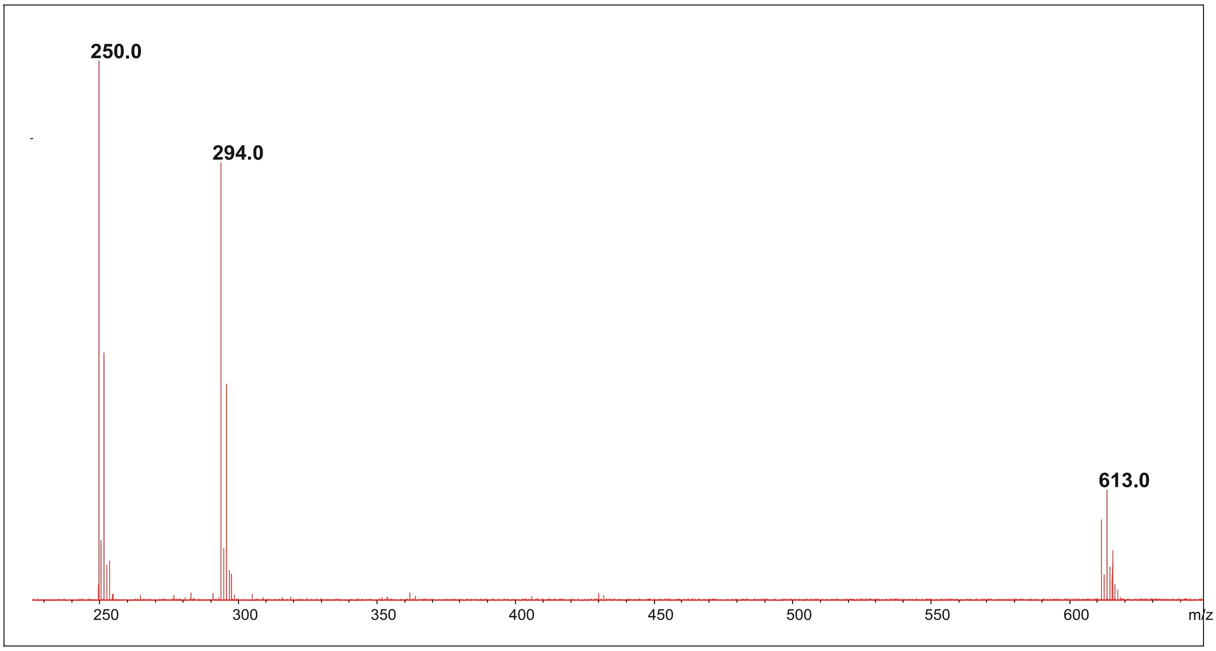

Figure S6. Fragment of the ESI-MS(−) plot of **1**.

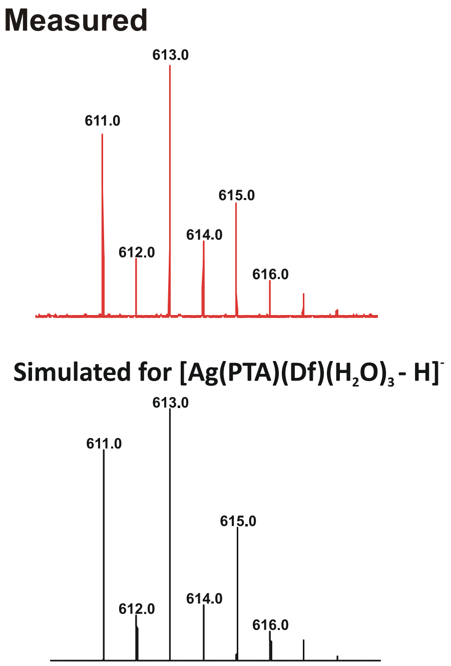

Figure S7. Experimental and simulated isotopic distribution pattern for $[Ag(PTA)(Df)(H_2O)_3 - H]^-$ ESI-MS(−) of **1**.

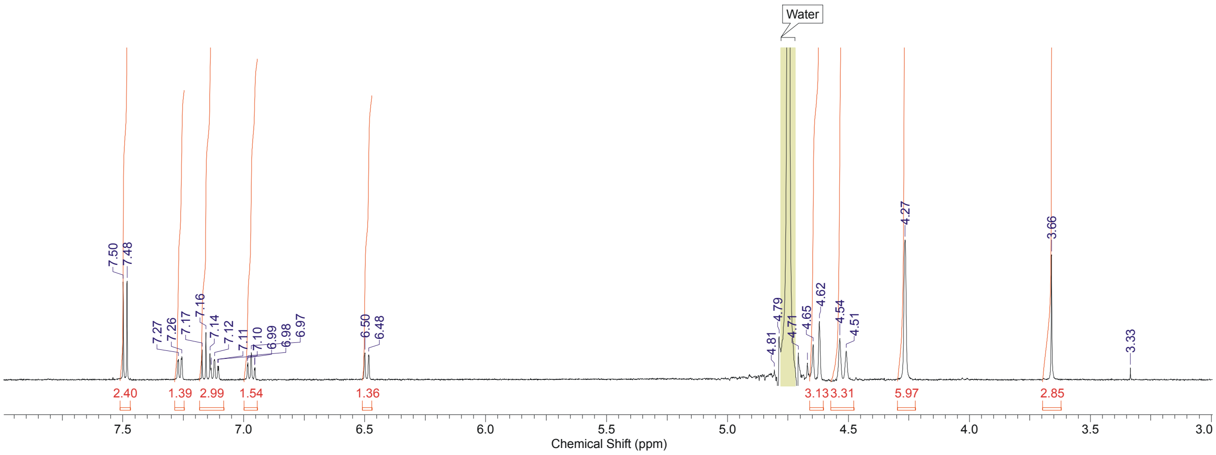

Figure S8. ^{1}H NMR spectrum of **1** in D_2O.

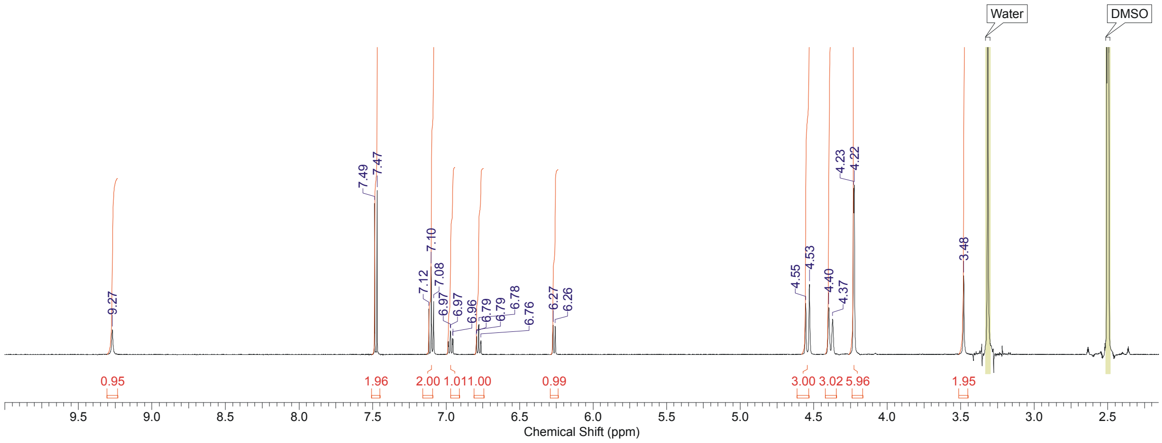

Figure S9. ^{1}H NMR spectrum of **1** in DMSO-d_6.

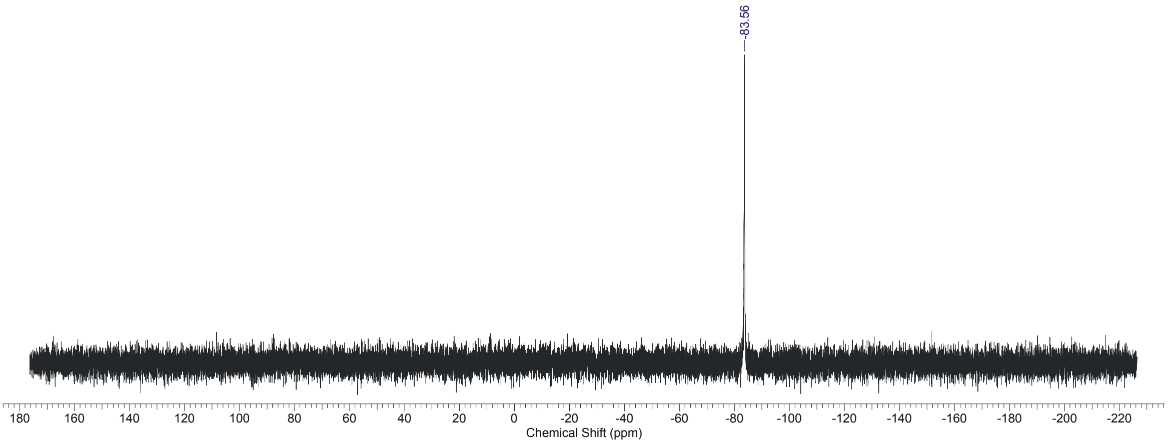

Figure S10. $^{31}P[^{1}H]$ NMR spectrum of **1** in DMSO-d_6.

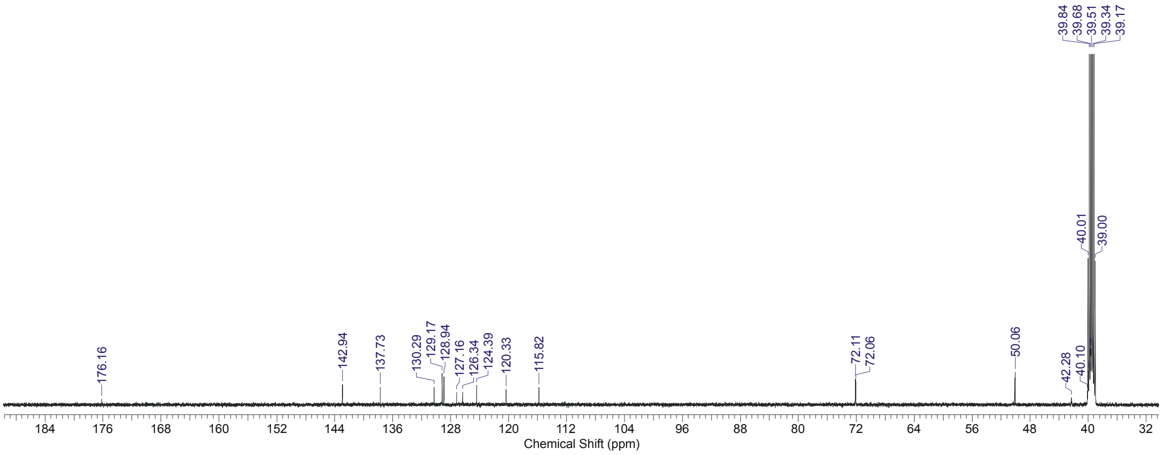

Figure S11. $^{13}C[^{1}H]$ NMR spectrum of **1** in DMSO-d_6.

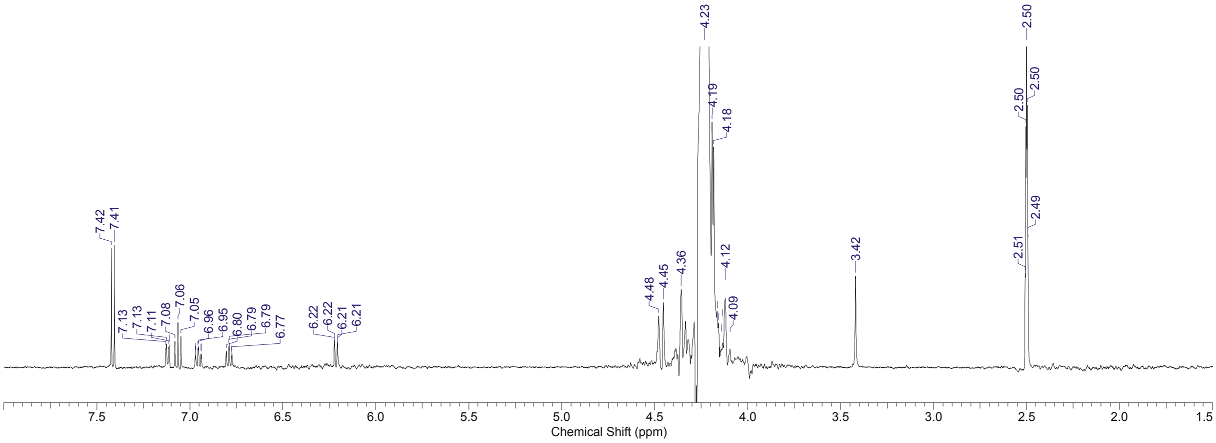

Figure S12. ^{1}H NMR spectrum of **1** in DMSO-d_6/D_2O = 5/2.

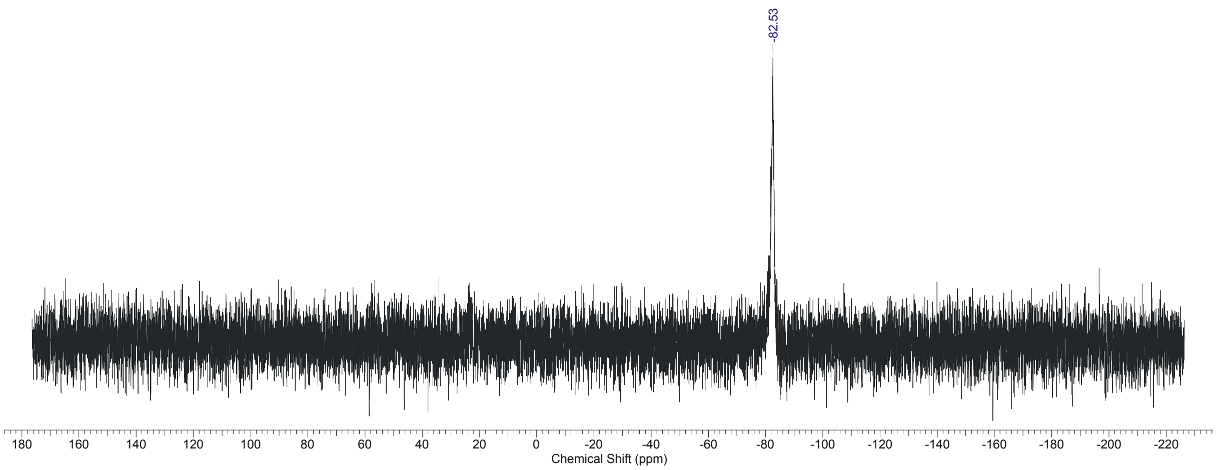

Figure S13. $^{31}P[^{1}H]$ NMR spectrum of **1** in DMSO-d_6/D_2O = 5/2.

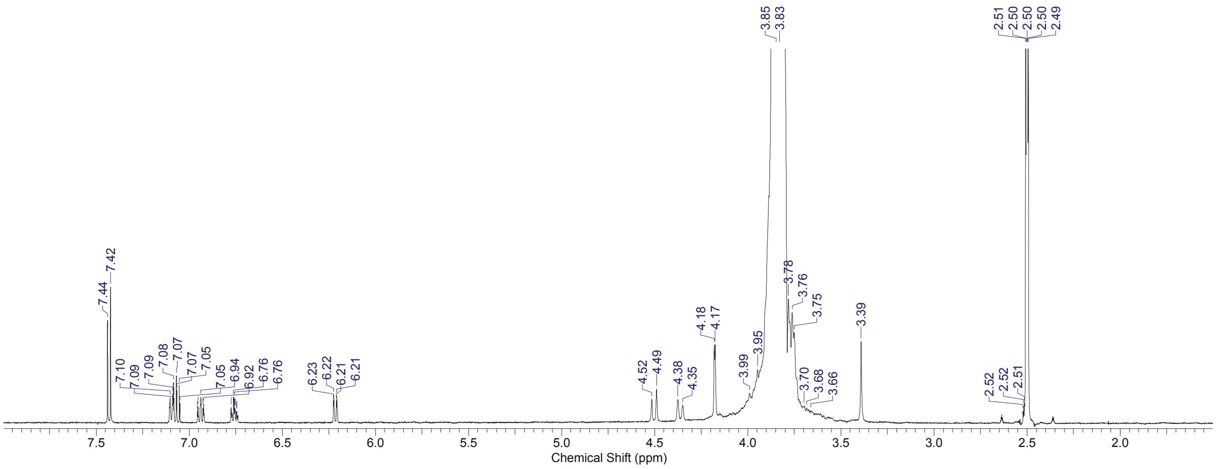

Figure S14. ^{1}H NMR spectrum of **1** in DMSO-d_6/D_2O = 5/2 in presence of NaCl (c=5 mM).

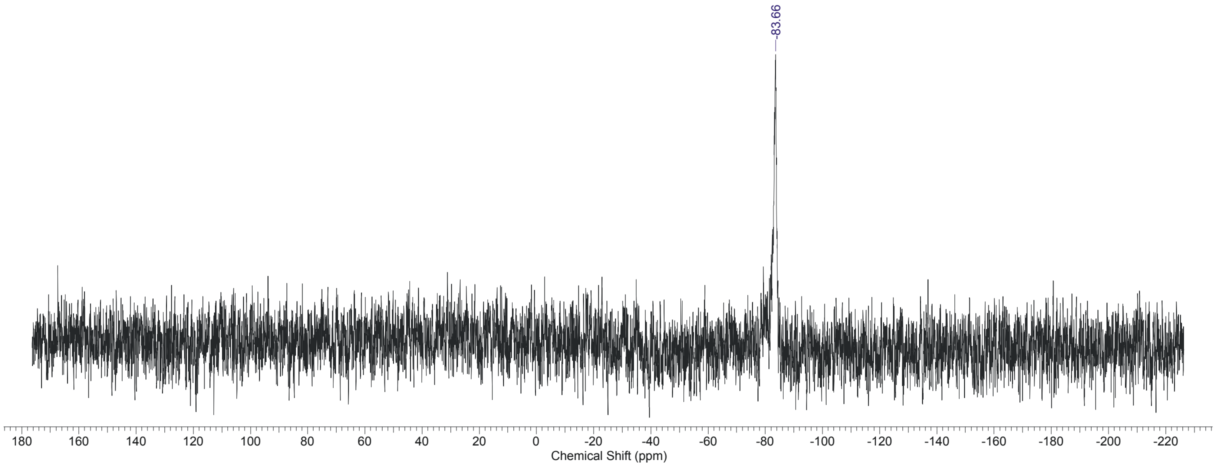

Figure S15. $^{31}P[^{1}H]$ NMR spectrum of **1** in DMSO-d_6/D_2O = 5/2 in presence of NaCl (c=5 mM).

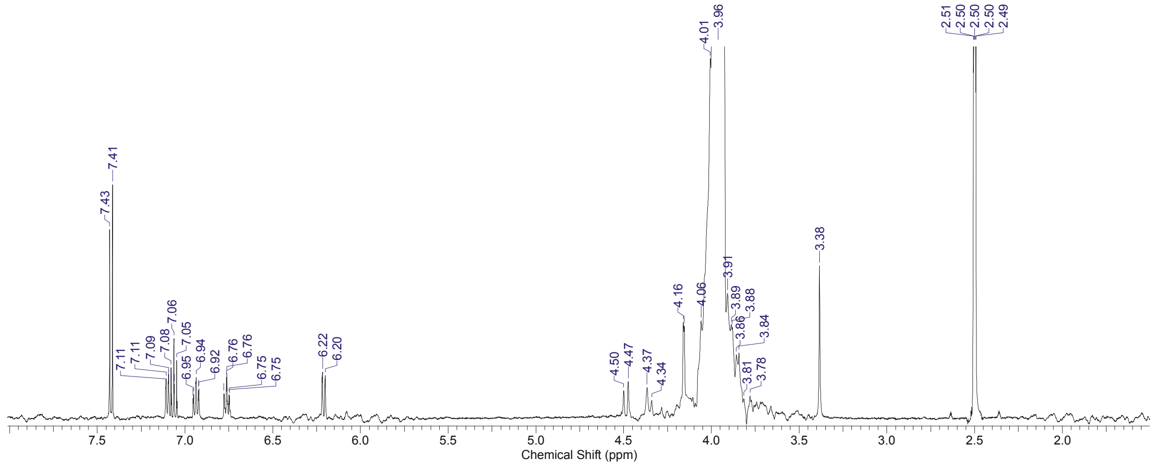

Figure S16. ^{1}H NMR spectrum of **1** in DMSO-d_6/D_2O = 5/2 in presence of NaCl (c=50 mM).

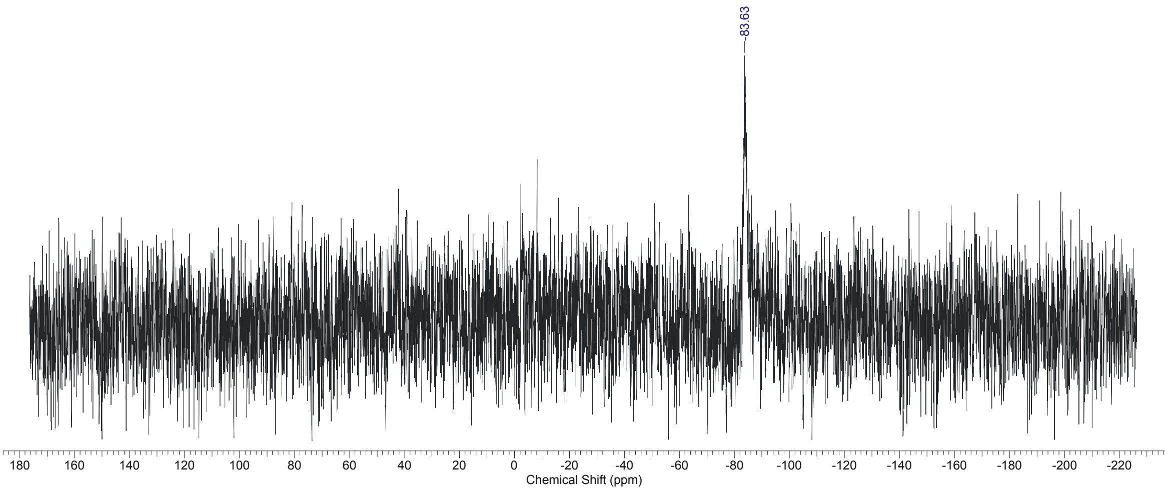

Figure S17. ^{31}P[^{1}H] NMR spectrum of **1** in DMSO-d_6/D_2O = 5/2 in presence of NaCl (c=50 mM).

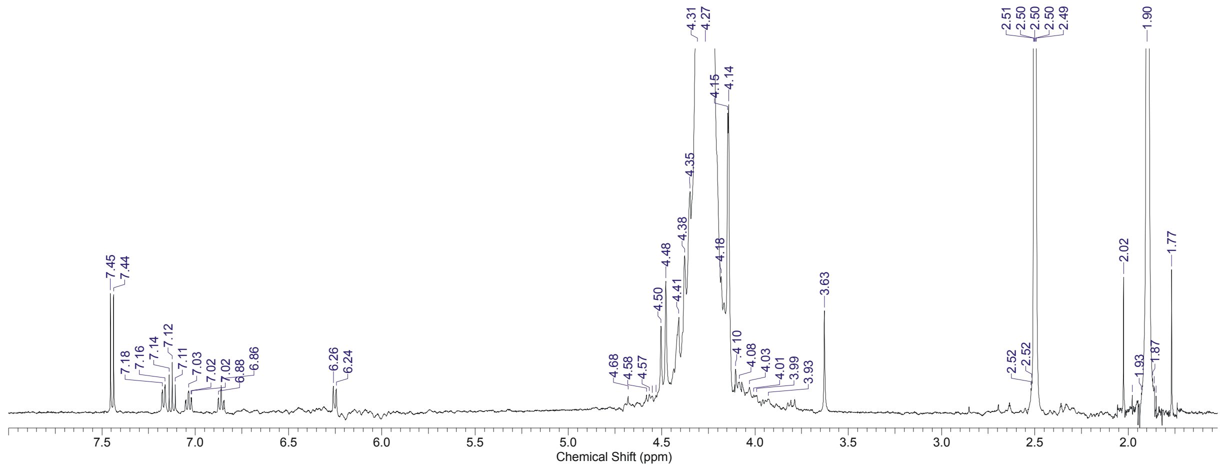

Figure S18. ^{1}H NMR spectrum of **1** in DMSO-d_6/D_2O = 5/2 in presence of acetic acid buffer (pH=5.5).

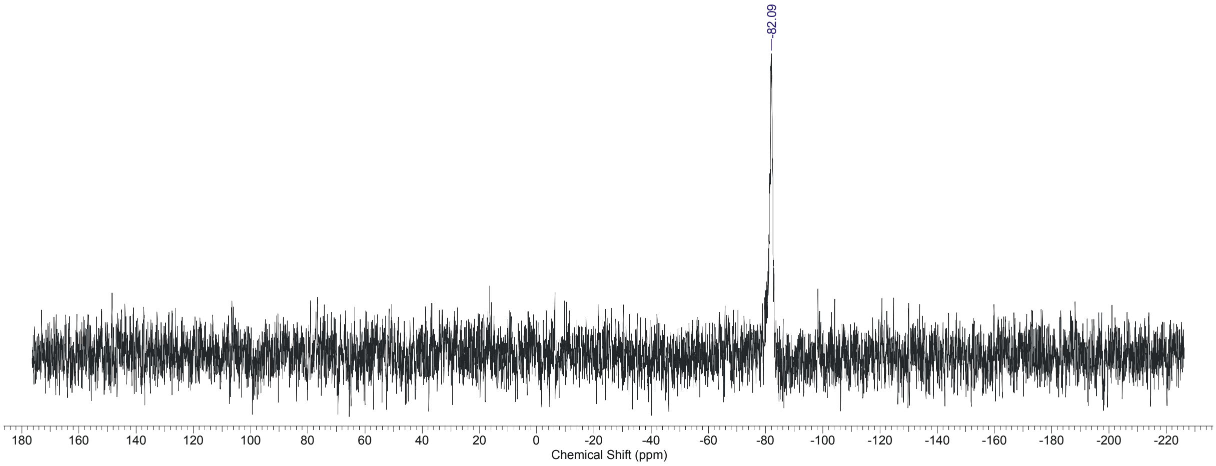

Figure S19. $^{31}P[^{1}H]$ NMR spectrum of **1** in DMSO-d_6/D_2O = 5/2 in presence of acetic acid buffer (pH=5.5).

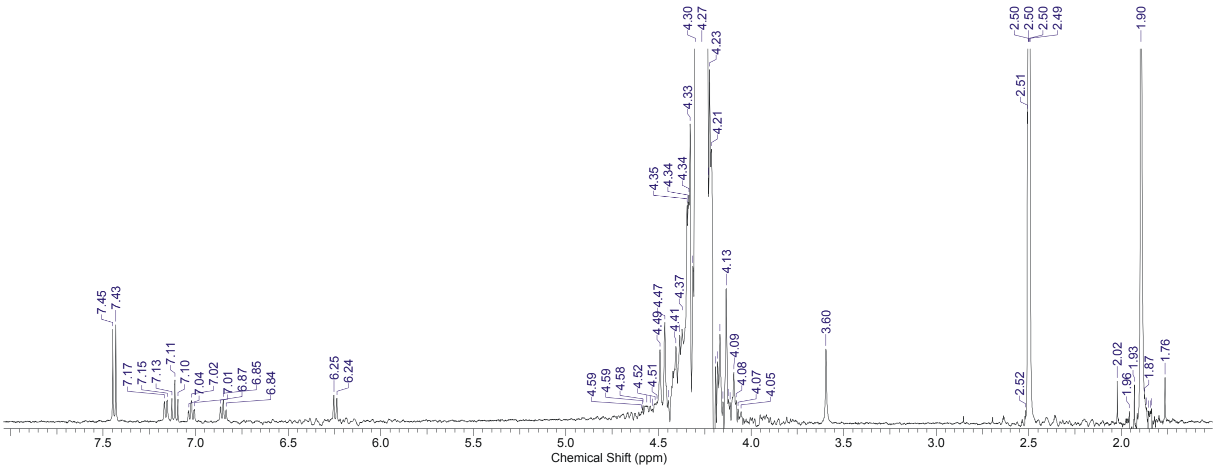

Figure S20. ^{1}H NMR spectrum of **1** in DMSO-d_6/D_2O = 5/2 in presence of acetic acid buffer (pH=4.0).

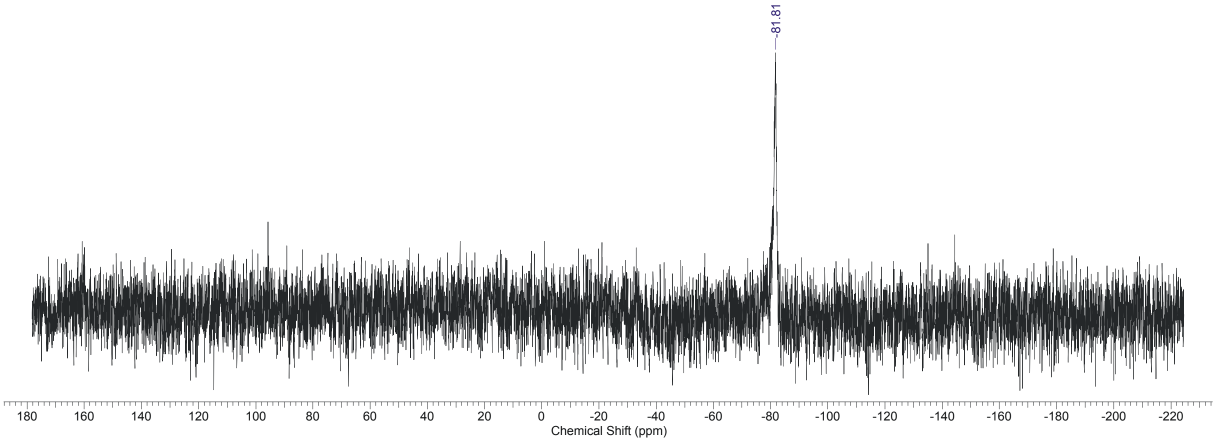

Figure S21. $^{31}P[^{1}H]$ NMR spectrum of **1** in DMSO-d_6/D_2O = 5/2 in presence of acetic acid buffer (pH=4.0).

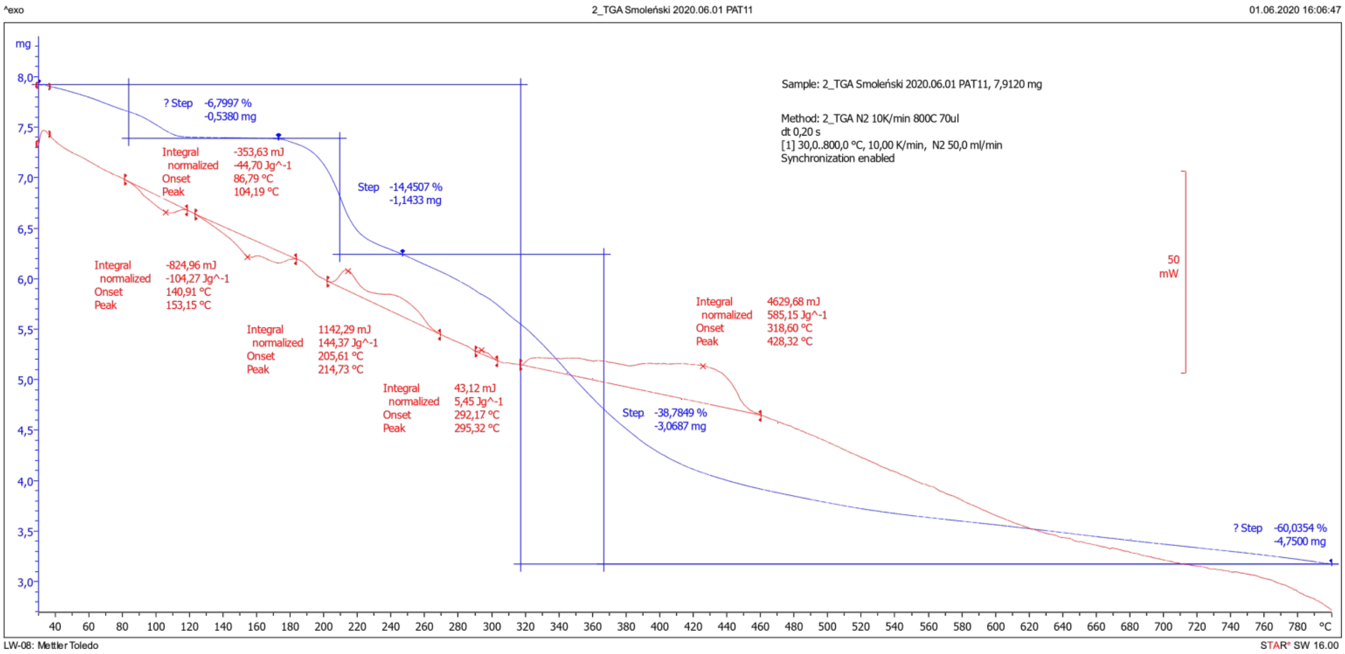

Figure S22. TGA analysis of **1**.

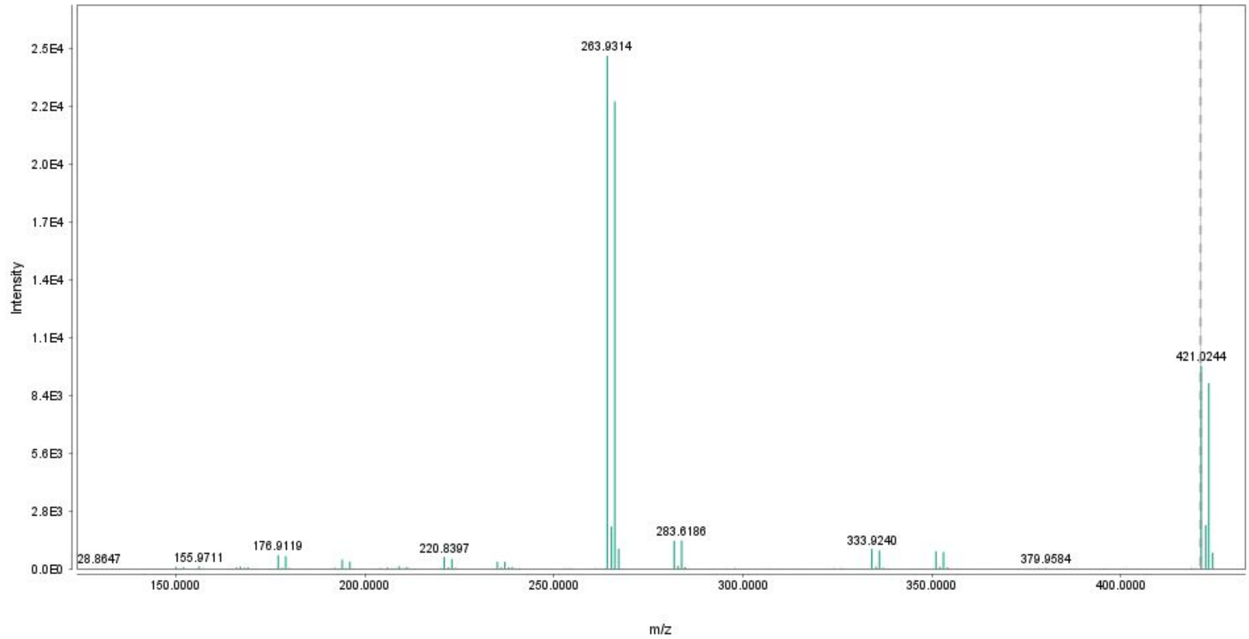

Figure S23. Positive ESI-MS^2 plot of cation $[Ag(PTA)_2]^+$ fragment of **1**.

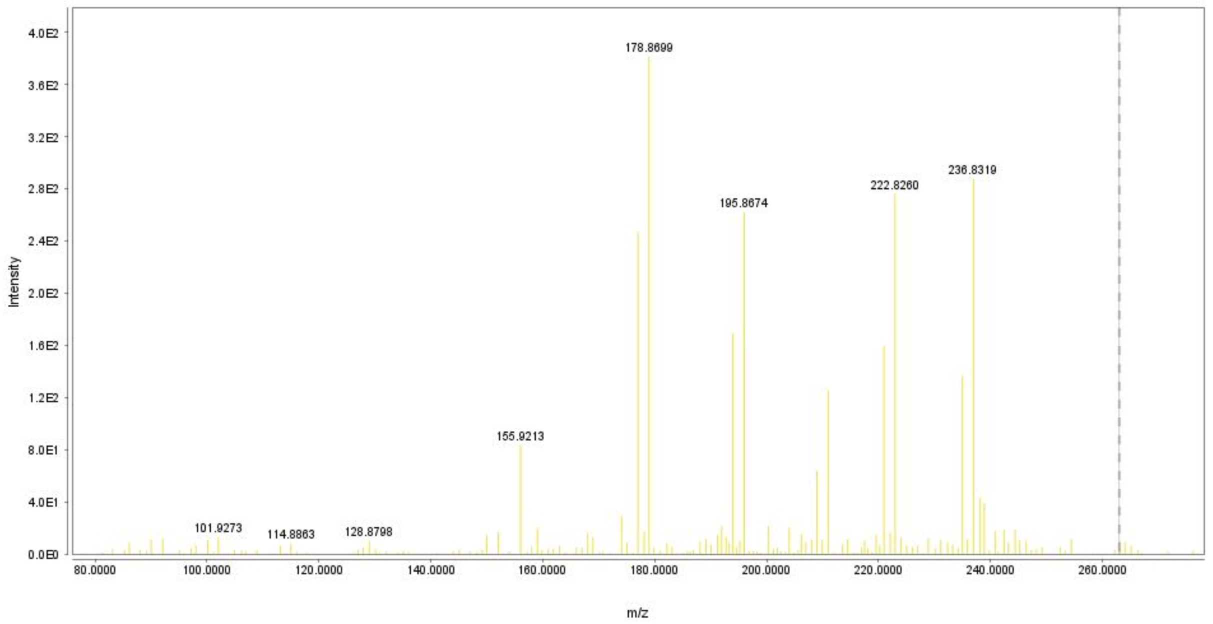

Figure S24. Positive ESI-MS^3 plot of cation $[Ag(PTA)_2]^+$ fragment of **1**.

Supplementary Table

Table S1. Results of NMR tests in presence of NaCl and acetic acid buffer: Chemical shifts of functional groups in ^{1}H NMR and $^{31}P[^{1}H]$ NMR spectra of **1** in D_2O, DMSO-d_6, mixture of in DMSO-d_6/D_2O=5/2, and in the presence of NaCl (5 and 50 mM) and acetic acid buffer (pH=4.0 and 5.5).

**Solvents/ Conditions**	**δ ^{1}H NMR, C_6H_3, Df, ppm**	**δ ^{1}H NMR, C_6H_4, Df, ppm**	**δ ^{1}H NMR, NCH_AH_BN, PTA, ppm**	**δ ^{1}H NMR, PCH_2N, PTA, ppm**	**δ ^{1}H NMR, CH_2COO, Df, ppm**	**δ $^{31}P[^{1}H]$ NMR, ppm**	**Spectrum No.**
D_2O	7.49, 7.15	7.26, 7.12, 6.97, 6.49	4.58	4.27	3.66	-	S4
DMSO-d_6	7.48, 7.10	7.09, 6.97, 6.49, 6.27	4.65	4.23	3.48	-83.6	S5<br>S6
DMSO-d_6/D_2O=5/2	7.41, 7.06	7.13, 6.95, 6.79, 6.22	4.39	4.18	3.42	-82.5	S8<br>S9
DMSO-d_6/D_2O=5/2;<br>5 mM NaCl	7.43, 7.07	7.09, 6.94, 6.76, 6.21	4.44	4.18	3.39	-83.7	S10<br>S11
DMSO-d_6/D_2O=5/2;<br>50 mM NaCl	7.42, 7.06	7.10, 6.94, 6.76, 6.21	4.42	4.16	3.38	-83.6	S12<br>S13
DMSO-d_6/D_2O=5/2;<br>Acetic acid buffer pH=5.5	7.44, 7.12	7.17, 7.03, 6.86, 6.25	4.43	4.15	3.63	-82.1	S14<br>S15
DMSO-d_6/D_2O=5/2;<br>Acetic acid buffer pH=4.0	7.44, 7.11	7.16, 7.02, 6.85, 6.25	4.43	4.23	3.60	-81.8	S16<br>S17

Table S2. Statistical significance of percentage dependence of viable cells (Annexin V-(−)), early apoptosis (Annexin V(+) and PI(−)), late apoptosis (Annexin V(+) and PI(-)), and necrosis (Annexin V(−) and PI(+)). Data are presented as mean±SD from at least three independent biological experiments.

	Live	Apoptosis	Late Apoptosis	Necrosis
0.1	*	ns	ns	ns
1	***	ns	ns	ns
5	***	***	*	ns
10	***	***	ns	ns
50	***	***	*	ns
100	***	***	***	**
500	***	***	***	***

	A549		PANC-1	
Live		***		***
Apoptosis	ns	***	ns	*
Late apoptosis	ns	*	ns	***
Necrosis	ns	ns	ns	***

Table S3. Surviving fractions (%) estimated relative to untreated controls of selected cells.[a]

		**A549**	**MCF-7**	**Du-145**	**PANC-1**	**HaCaT**
**6h + 24h**						
Diclofenac	dil. 100x	72.5 ± 6.8	78.6 ± 12.3	78.2 ± 5.6	75.3 ± 6.4	86.5 ± 11.7
	**dil. 1000x**	**88.7** ± 10.8	**89.5** ± 9.8	**87.7** ± 9.8	**84.5** ± 9.8	**92.5** ± 9.1
	dil. 10 000x	99.7 ± 4.3	102.6 ± 13.4	99.5 ± 8.2	98.6 ± 7.8	99.6 ± 10.8
PTA	dil. 100x	77.5 ± 9.8	78.6 ± 13.1	78.4 ± 11.8	74.7 ± 9.8	99.7 ± 4.9
	**dil. 1000x**	**82.4** ± 12.9	**89.6** ± 4.8	**89.3** ± 8.8	**86.5** ± 6.4	**112.7** ± 8.8
	dil. 10 000x	96.8 ± 10.8	88.6 ± 7.5	106.9 ± 9.2	89.7 ± 12.5	109.7 ± 6.3
$AgNO_3$	**dil. 1000x**	48.9 ± 6.5	62.7 ± 6.3	62.87 ± 5.9	65.89 ± 4.6	76.7 ± 6.8
	dil. 10 000x	68.1 ± 6.2	59.2 ± 8.2	74.5 ± 8.8	76.2 ± 7.4	88.1 ± 4.2
**24h + 24h**						
Diclofenac	dil. 100x	70.9 ± 8.9	73.7 ± 8.7	78.7 ± 7.6	71.2 ± 6.5	88.8 ± 5.8
	**dil. 1000x**	**81.8** ± 6.8	**88.7 ± 10.6**	**88.4 ± 6.7**	**82.4** ± 7.7	**99.2** ± 6.8
	dil. 10 000x	98.5 ± 10.9	98.2 ± 9.8	99.5 ± 10.7	89.5 ± 9.9	99.3 ± 12.8
PTA	dil. 100x	77.2 ± 12.8	70.9 ± 8.8	73.8 ± 12.6	78.7 ± 8.3	92.2 ± 5.3
	**dil. 1000x**	**88.2** ± 8.7	**82.7 ± 11.5**	**89.2 ± 5.8**	**85.2 ± 9.5**	**96.7 ± 9.7**
	dil. 10 000x	98.6 ± 5.9	100.2 ± 6.6	99.2 ± 7.2	107.5 ± 6.8	98.1 ± 12.1
$AgNO_3$	**dil. 1000x**	31.2 ± 5.5	38.5 ± 7.3	36.7 ± 7.1	44.2 ± 9.2	41.6 ± 6.9
	dil. 10 000x	57.4 ± 3.8	62.1 ± 5.5	58.8 ± 7.3	65.8 ± 6.6	72.7 ± 8.1
**72h + 24h**						
Diclofenac	dil. 100x	67.7 ± 6.5	71.6 ± 9.9	70.8 ± 7.9	68.5 ± 6.9	88.4 ± 5.2
	**dil. 1000x**	**78.5** ± 7.6	**85.4 ± 5.3**	**71.4 ± 8.5**	**80.3 ± 4.1**	**99.2 ± 5.9**
	dil. 10 000x	89.5 ± 8.2	97.8 ± 7.7	88.7 ± 6.1	89.4 ± 6.3	102.3 ± 11.2
PTA	dil. 100x	67.3 ± 8.6	84.7 ± 8.9	62.3 ± 8.7	70.8 ± 6.7	91.2 ± 6.5
	**dil. 1000x**	**79.8 ± 7.5**	**88.6 ± 7.9**	**84.2 ± 8.2**	**83.4 ± 5.1**	**98.2 ± 8.4**
	dil. 10 000x	92.7 ± 8.6	102.5 ± 6.0	101.2 ± 12.4	98.9 ± 8.9	100.6 ± 8.1
$AgNO_3$	**dil. 1000x**	34.1 ± 5.3	36.0 ± 8.4	42.8 ± 5.9	55.4 ± 6.7	65.2 ± 6.3
	dil. 10 000x	55.8 ± 8.1	56.8 ± 6.2	56.1 ± 5.9	65.8 ± 6.1	70.5 ± 5.8

[a]Details of the experiments performed: A549, MCF-7, Du-145, PANC-1, and HaCaT cells were treated with diclofenac (0.918 mg/mL), PTA (0.483 mg/mL), and $AgNO_3$ (0.326 mg/mL) diluted 100, 1 000, 10 000 times in appropriate medium with 1% FBS. Three different experimental approaches were carried out with different incubation times with the relevant compound - 6, 24, and 72 h. After each incubation time, the cells were washed with PBS. Then, fresh medium (supplemented with 10% FSB) was added, and the cells were incubated under standard conditions for another 24 h (6h + 24h, 24h + 24h, 72h + 24h). After this time, the MTT survival test was done. Surviving fraction given in % was estimated with relation to untreated cells. In the case of 6h + 24h and 24h + 24h approaches, the cells were seeded at 10 000 cells per well, while in the case of 72h + 24h at 5 000 cells/well.

Reference

S1. Komarnicka, U. K.; Kozieł, S.; Zabierowski, P.; Kruszyński, R.; Lesiów, M. K.; Tisato, F.; Porchia, M.; Kyzioł, A. Copper(I) complexes with phosphines P(p-OCH_3-Ph)$_2$$CH_2$OH and P(p-$OCH_3$-Ph)$_2$$CH_2$SarGly. Synthesis, multimodal DNA interactions, and prooxidative and in vitro antiproliferative activity. *J. Inorg. Biochem*. **2020**, *203*, 110926.
